# The Lys‐motif receptor *LYK4* mediates *Enterobacter* sp. SA187 triggered salt tolerance in *Arabidopsis thaliana*

**DOI:** 10.1111/1462-2920.15839

**Published:** 2021-12-23

**Authors:** Eleonora Rolli, Axel de Zélicourt, Hanin Alzubaidy, Michael Karampelias, Sabiha Parween, Naganand Rayapuram, Baoda Han, Katja Froehlich, Aala A. Abulfaraj, Hanna Alhoraibi, Kiruthiga Mariappan, Cristina Andrés‐Barrao, Jean Colcombet, Heribert Hirt

**Affiliations:** ^1^ Université Paris‐Saclay, CNRS, INRAE, Université d'Evry, Université de Paris, Institute of Plant Sciences Paris‐Saclay (IPS2) Orsay France; ^2^ DARWIN21, Center for Desert Agriculture King Abdullah University of Science and Technology Thuwal Saudi Arabia; ^3^ Department of Biological Sciences, Science and Arts College, Rabigh Campus King Abdulaziz University Jeddah Saudi Arabia; ^4^ Department of Biochemistry, Faculty of Science King Abdulaziz University Jeddah Saudi Arabia; ^5^ Max F. Perutz Laboratories University of Vienna Vienna Austria; ^6^ Present address: Department of Food, Environmental and Nutritional Sciences (DeFENS) University of Milan, via Celoria 2 Milan 20133 Italy; ^7^ Present address: Red Sea Research Center King Abdullah University of Science and Technology Thuwal Saudi Arabia

## Abstract

Root endophytes establish beneficial interactions with plants, improving holobiont resilience and fitness, but how plant immunity accommodates beneficial microbes is poorly understood. The multi‐stress tolerance‐inducing endophyte *Enterobacter* sp. SA187 triggers a canonical immune response in Arabidopsis only at high bacterial dosage (>10^8^ CFUs ml^−1^), suggesting that SA187 is able to evade or suppress the plant defence system at lower titres. Although SA187 flagellin epitopes are recognized by the *FLS2* receptor, SA187‐triggered salt tolerance functions independently of the *FLS2* system. In contrast, overexpression of the chitin receptor components *LYK4* and *LYK5* compromised the beneficial effect of SA187 on Arabidopsis, while it was enhanced in *lyk4* mutant plants. Transcriptome analysis revealed that the role of *LYK4* is intertwined with a function in remodelling defence responses with growth and root developmental processes. *LYK4* interferes with modification of plant ethylene homeostasis by *Enterobacter* SA187 to boost salt stress resistance. Collectively, these results contribute to unlock the crosstalk between components of the plant immune system and beneficial microbes and point to a new role for the Lys‐motif receptor *LYK4* in beneficial plant–microbe interaction.

## Introduction

The rhizosphere, a narrow zone of nutrient‐rich soil that surrounds and is influenced by plant roots, is densely colonized by a plethora of microorganisms, including fungi, bacteria, protists, nematodes and invertebrates (Delgado‐Baquerizo *et al*., 2016; Pascale *et al*., 2020). At a significant cost of carbon and nitrogen for the plant (Pausch and Kuzyakov, 2018), the root system releases a vast array of primary and specialized metabolites into the rhizosphere (Olanrewaju *et al*., 2019), influencing the root chemistry (Rolfe *et al*., 2019) to recruit, repel, shape, signal or interfere with the rhizosphere microbiome (Liu *et al*., 2021), with the ultimate benefit of promoting beneficial microorganisms (Rolli *et al*., 2021) while combating pathogenic ones (Venturi and Keel, 2016). The plant microbiome can act as a critical extension of the plant phenotype (Berendsen *et al*., 2012; Zhang *et al*., 2021) to help in the uptake of nutrients (Dhawi *et al*., 2015; Babalola *et al*., 2020), in enhancing stress tolerance (Marasco *et al*., 2012; Rolli *et al*., 2015; Liu *et al*., 2020), or in protecting plants against pathogens, through antagonism and competition or by stimulating the plant immune system (Berendsen *et al*., 2012; Vannier *et al*., 2019). The plant immune system represents the biological barrier that detects and discriminates among invaders, symbiotic or mutualistic microorganisms (Cook *et al*., 2015; Nishad *et al*., 2020).

Microorganisms possess microbe‐associated molecular patterns (MAMPs), like flagellin, elongation factor Tu (EF‐Tu), cold shock protein, lipopolysaccharide (LPS) or chitin (Boutrot and Zipfel, 2017), which are perceived and discriminated by pattern recognition receptors (PRRs) on the plant cell surface. Once activated, PRRs recruit and form complexes with receptor‐like cytoplasmic kinases to trigger MAMP‐triggered immunity (MTI), which can be efficiently induced against potential pathogens within minutes after activation. H^+^ and Ca^2+^ fluxes as well as the production of reactive oxygen species (ROS) are among the earliest responses to MAMP detection (Bigeard *et al*., 2015). Mediated by Ca^2+^‐dependent protein kinases and mitogen‐activated protein kinases (MAPKs), these signalling cascades eventually trigger the transcriptional regulation of defence related genes, leading to callose deposition, antimicrobial compound synthesis and defence hormone regulation, like salicylic acid (SA), ethylene and jasmonic acid (JA) (Nishad *et al*., 2020). The plant immune response has been extensively investigated in the context of recognition of plant pathogens. However, the complex interplay occurring during beneficial interaction is less understood, although attracting increasing interest due to the role played by the beneficial microbiome for holobiont fitness (Voolstra and Ziegler, 2020). Furthermore, only a modest array of studies investigates the plant immune modulation in the context of abiotic stress to favour the recruitment of beneficial bacteria able to sustain plant growth under adverse environmental conditions (Cheng *et al*., 2019; Saijo and Loo, 2020). Successful interactions need to stimulate symbiotic signalling pathways, as in the case of the nitrogen‐fixing symbiosis with rhizobia or mycorrhizal fungi (Wang *et al*., 2018) or to evade and suppress plant immune responses by free‐living plant growth promoting bacteria (Zamioudis and Pieterse, 2012).

Different escaping strategies have been documented for beneficial bacteria. For instance, in grapevine, the *FLS2* receptor differentially recognizes Flg22 epitopes derived from beneficial *Paraburkholderia phytofirmans* strain PsJN compared with those produced by the pathogenic bacteria *Pseudomonas aeruginosa* strain PAK and *Xanthomonas campestris* pv. *campestris*, inducing a reduced immune response (Trdá *et al*., 2014). Recent reports documented that *Pseudomonas simiae* WCS417 and *Bacillus subtilis* FB17 can actively suppress root immune responses (Millet *et al*., 2010; Lakshmanan *et al*., 2012; Stringlis *et al*., 2018): *P*. *simiae* WCS417 produces organic acids that decrease the environmental pH, thus suppressing immune defence (Yu *et al*., 2019), while *B*. *subtilis* FB17 acts through a JA‐dependent mechanism (Lakshmanan *et al*., 2012).

The present work investigates the interplay between Arabidopsis immune system and the beneficial endophytic bacterium *Enterobacter* sp. SA187 (de Zélicourt *et al*., 2018; Shekhawat *et al*., 2021) in the context of salt stress. The early events of interaction were investigated to evaluate whether the bacterium adopts escape strategies and we observed that plant immunity reactions to SA187 inoculation depend on the microbial concentrations to which the plant is exposed to. For titres ≤10^6^ CFUs ml^−1^, SA187 escaped plant immune detection and did not activate defence responses, while treatment with higher bacterial concentrations (≥10^8^ CFUs ml^−1^) triggered canonical immune responses that were not suppressed upon colonization (24 h). The five flagellin genes encoded by SA187 are all expressed during the interaction with the host plant and the immunogenic peptides induced canonical MAMP‐associated reactions. Furthermore, the involvement of the plant immune system in SA187‐triggered salt resistance was analysed by verifying the ability of the bacterium to support plant growth under saline conditions in several immune receptor mutants. Intriguingly, the chitin receptor *LYK4* and, to a minor degree, *LYK5* (Wan *et al*., 2012; Xue *et al*., 2019) act as negative regulators of SA187‐triggered salt stress resistance. Through phenotypic and transcriptomic approaches, we observed that *LYK4* affects SA187‐enhanced salt resistance by regulating plant defence responses, thus affecting growth and development. These data reveal that the immune receptor *LYK4* is tightly involved in the coordination of the plant immune response with the beneficial effects of SA187 to induce salt stress resistance.

## Results

### *Enterobacter* sp. SA187 triggers plant immunity responses

**Fig. 1:**
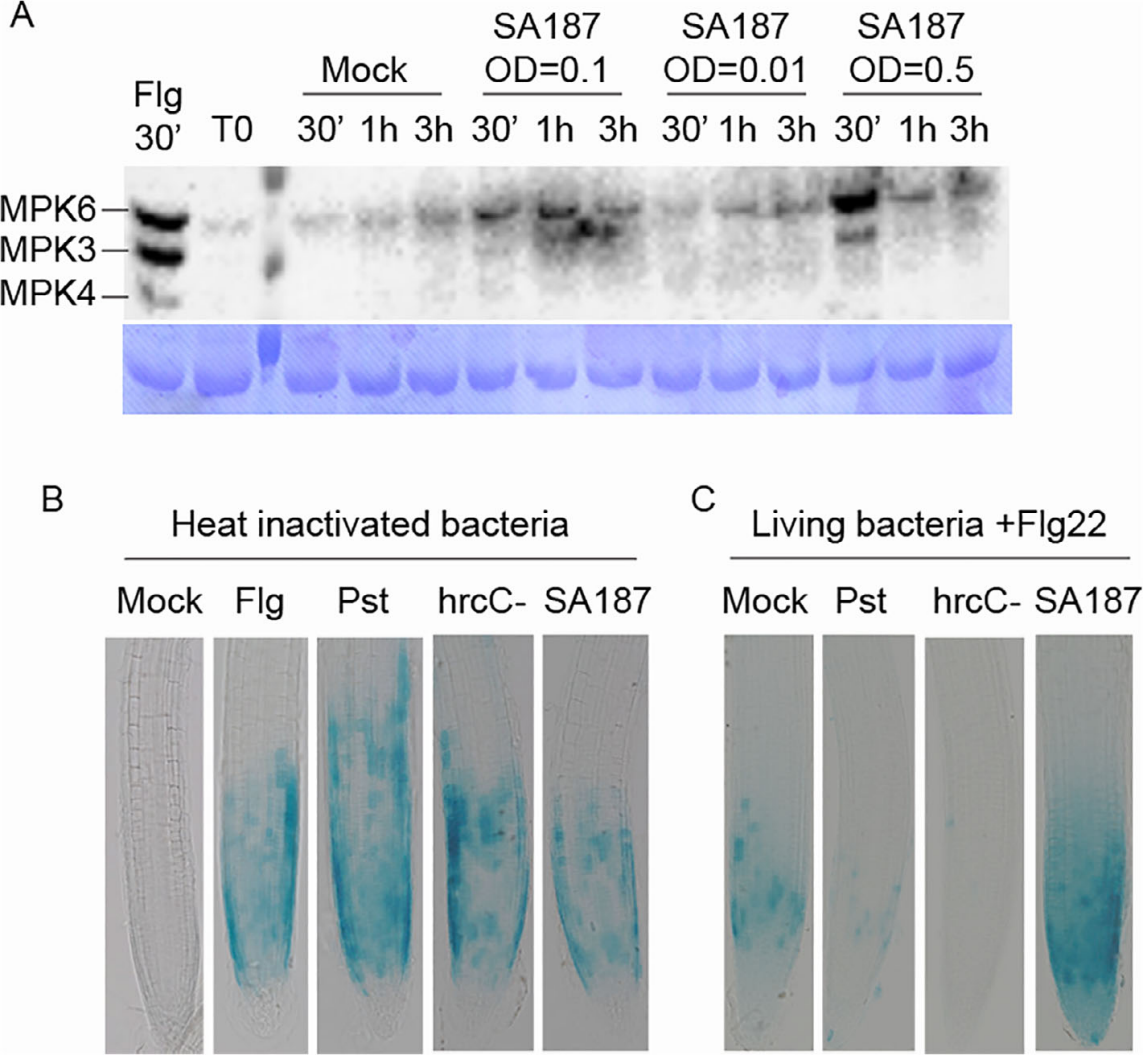
*Enterobacter* sp. SA187 induced canonical plant immune response. A. Typical MAPK activation in Col‐0 seedlings after treatment with 1 μM flagellin peptide or H_2_O (mock) or SA187 bacterial suspensions at different concentrations: 0.5 OD, 0.1 OD and 0.01 OD, corresponding respectively to 6 × 10^8^, 1 × 10^8^ and 7 × 10^6^ CFUs ml^−1^. The three detected MAPKs were identified as MAPK6, MAPK3 and MAPK4 by comparison with the positive control of plantlets treated with 1 μM flagellin peptide (Flg22) and are indicated in the figure. Coomassie staining of the membrane was adapted to show similar protein loading in each well. M = protein marker. B. GUS staining of the reporter line *pCYP71A12*::*GUS* upon treatment for 5 h with 10 μM Flg22 or heat inactivated bacteria (OD = 0.1) of *Pst* DC3000, *Pst* hrcC^−^ or SA187. C. GUS staining of the reporter line *pCYP71A12*::*GUS* that was colonized by *Pst* DC3000, *Pst* hrcC^−^ or SA187 for 10 days after germination and then treated for 5 h with 10 μM Flg22.

To assess whether *Enterobacter* sp. SA187 could lead to MTI responses, 11 days old Arabidopsis plantlets were exposed to three different SA187 dosages (Supporting Information Fig. S1A) and MAPK cascade activation was investigated as key event in immune signalling after MAMP perception. MAPK activation was monitored by western blot analysis using the anti‐pTpY antibody, which recognizes the phosphorylated forms of the three canonical MAPKs – MPK3, MPK4 and MPK6 – as a proxy of their activation (Fig. 1A). MAPK3 and MAPK6 were activated in a dose‐dependent manner only at high cell density (OD = 0.5, corresponding to 6 × 10^8^ CFUs ml^−1^ and OD = 0.1, corresponding to 1 × 10^8^ CFU ml^−1^). In contrast, at OD = 0.01, corresponding to 7 × 10^6^ CFUs ml^−1^, SA187 did not activate MAPKs (Fig. 1A). When SA187 was supplied at 6 × 10^8^ CFUs ml^−1^, MAPK activation peaked at 30 min, while the maximum activation level lasted 60 min when Arabidopsis was exposed to a bacterial density of 1 × 10^8^ CFUs ml^−1^ (Fig. 1A). These results suggest that SA187 is recognized in a dose‐dependent manner through the plant immune system.

To determine whether Arabidopsis roots respond to MAMPs expressed by SA187, a transgenic Arabidopsis line, which expressed the GUS reporter gene under the *CYP71A12* promoter, was used. *CYP71A12* encodes a cytochrome P450 that contributes to camalexin biosynthesis (Nafisi *et al*., 2007) and is upregulated in response to flagellin (Denoux *et al*., 2008). The GUS reporter gene was induced similarly to Flg22 (10 μM) in the root meristematic and elongation zone by heat‐killed bacterial SA187 cells (OD = 0.1) in a similar fashion to heat‐killed bacterial cells of the pathogen *Pseudomonas syringae* pv. tomato DC3000 (*Pst*) or the *Pst* mutant hrcC (*hrcC‐*), that lacks a functional the type III secretion system (T3SS) for injecting effectors into the host cells (Fig. 1B). This result indicates that SA187 possesses MAMPs similar to known pathogenic *Pseudomonas* strains and is able to induce analogous immunity reactions in roots. Surprisingly, colonization by living SA187 cells could not suppress the Flg22‐induced GUS expression by the *CYP71A12* promoter (Fig. 1C). Based on these observations, SA187 is recognized by the plant immune system and triggers typical Flg22‐like immune responses, but it is unable to suppress these upon colonization.

### SA187‐encoded flagellins trigger canonical MAMP‐induced responses

**Fig. 2:**
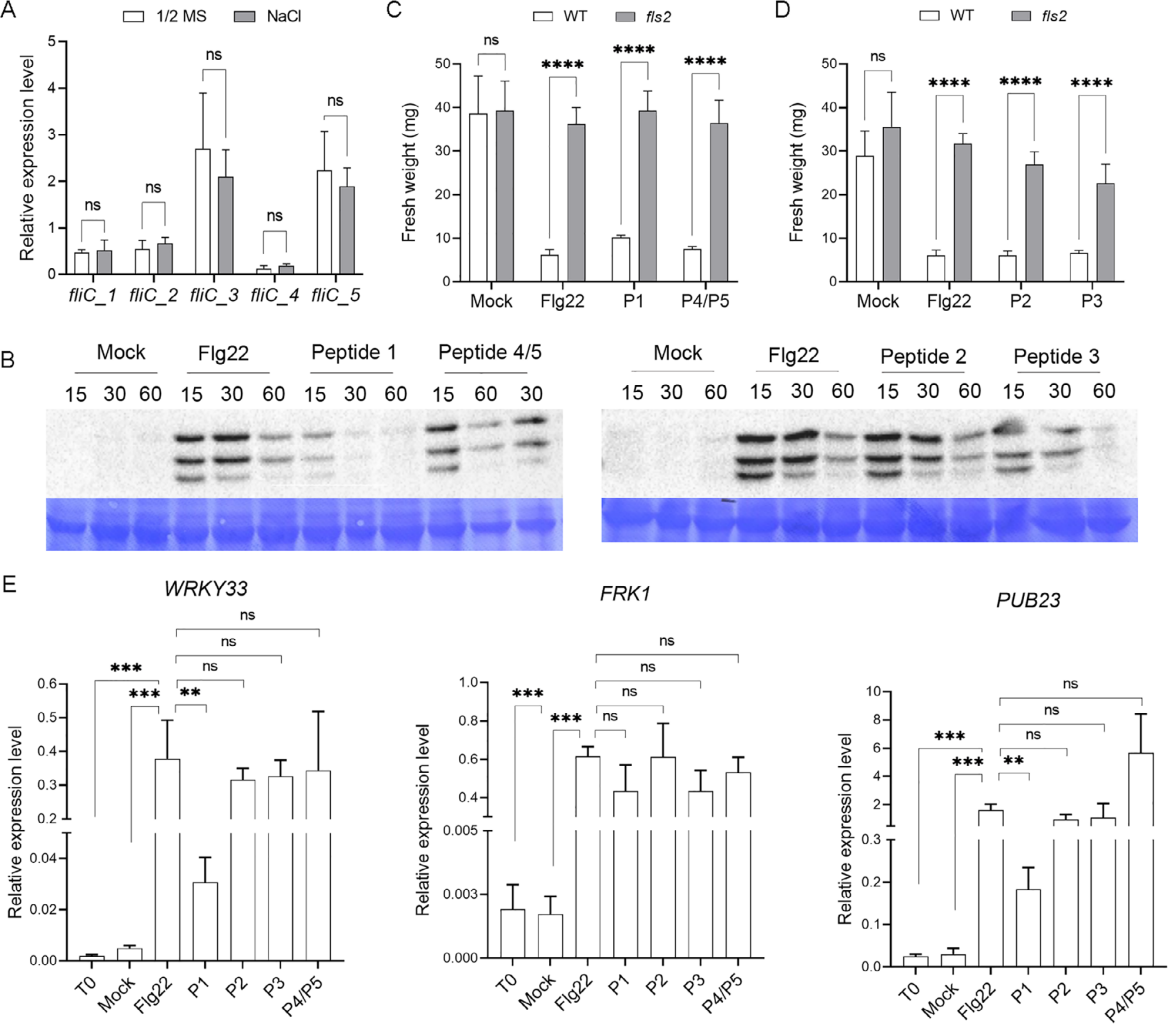
SA187‐encoded flagellins are endowed with MAMP features. A. SA187 encoded FliC genes expression in 14 day**‐**Arabidopsis colonized plantlets in 1/2 MS medium and 1/2 MS medium supplemented with 100 mM NaCl. Transcript accumulation is expressed as relative to the average of the transcript level of infB as reference gene, accordingly to Andrés‐Barrao *et al*. (2017). Bars represent average of three replicates ± SD. No difference in the relative expression level of each FliC gene was observed in the two assayed conditions according to two‐way ANOVA with Bonferroni's multiple comparison test (*P* ≥ 0.05). B. Time course MAPK activation in 11 days old Col‐0 seedlings after treatment with 100 nM of 22aa‐flagellin peptides encoded by SA187 in comparison to *Pseudomonas aeruginosa* Flg22 or mock inoculation for 15, 30 and 60 min. Coomassie staining of the membrane was adopted to show similar protein loading in each well. M = protein marker. C. Seedling growth inhibition assay. Five‐day‐old Col‐0 and *fls2* seedlings were transferred to liquid 1/2 MS supplemented or not with Flg22 and the different SA187‐encoded flagellin peptides at 1 μM concentration. Plants were weighted 7 days after treatment. Bars represent the average ± SD (*n* = 12). Statistical analysis was performed by applying the two‐way ANOVA with Bonferroni's test. ***P ≤ 0.001, n.s: statistically non‐significant. D. MAMP‐triggered immunity (MTI) marker genes induction in 11 days old Col‐0 seedlings at 60 min in response to 100 nM flagellin peptides. Transcript accumulation is expressed relative to the average of the transcript level of actin as reference gene. Bars represent average of three technical replicates ± SD. Results are representative of two independent experiments. Statistical analysis was performed by applying the unpaired *t*‐test, by comparing the relative expression of the target genes to the sample treated with Flg22. ***P ≤ 0.001, **P ≤ 0.01; n.s: statistically non‐significant.

To verify whether SA187 MAMPs can be differentially discriminated by the plant immune system, our attention was caught by the presence of five different *fliC* genes encoding different flagellin variants in the *Enterobacter* sp. SA187 genome (Andrés‐Barrao *et al*., 2017). The expression of the five *fliC* genes was evaluated in SA187 cells colonizing Arabidopsis plantlets for 14 days in the presence or absence of 100 mM NaCl. All five *fliC* genes were expressed at similar expression levels irrespective of the presence or absence of salt stress (Fig. 2A). The five SA187 FliC proteins contain in their N‐terminus an amino acid motif that is 60%–80% identical to the 22 amino acid Flg22 peptide recognized as a MAMP by Arabidopsis (Supporting Information Fig. S2A). As some pathogens and symbiotic bacteria carry mutations in their flagellin proteins to escape recognition by *FLS2* (Trdá *et al*., 2014), we wondered whether SA187 FliC amino acid differences also allow such evasion. Four SA187 *fliC*‐encoded Flg22 (SA187‐FliC) peptides, further referred to as P1, P2, P3 and P4/P5, were synthesized and tested for their biological activity in Arabidopsis. First, we treated separately plantlets with the four peptides at concentrations of 100 nM and 1 μM to test whether the three MAMP‐induced MAPKs were activated (Figs 2B and S2B). The kinetics and intensity of activation were similar for Flg22 and SA187‐FliC peptides, with the notable exception of P1 that induced a lower response (Figs 2B and S2B). In a seedling growth inhibition assay, the SA187 and Flg22 peptides also showed similar growth inhibitory activity that was fully dependent on *FLS2* integrity (Figs 2C and D and S2C). Then, the induction of typical MAMP‐responsive genes was assessed upon a 60 min treatment with 100 nM Flg22 or SA187‐FliC peptides. P2, P3 and P4/5 peptides showed a similar induction of immunity‐related genes as Flg22, while P1 induced the transcription of the marker genes to a much lower degree (Fig. 2E). Therefore, the Arabidopsis immune system recognizes most SA187‐FliC peptides in a similar way as Flg22.

### Among the MAMP receptor components, *LYK4* and *LYK5* mediate the beneficial effect of SA187 in Arabidopsis

**Fig. 3:**
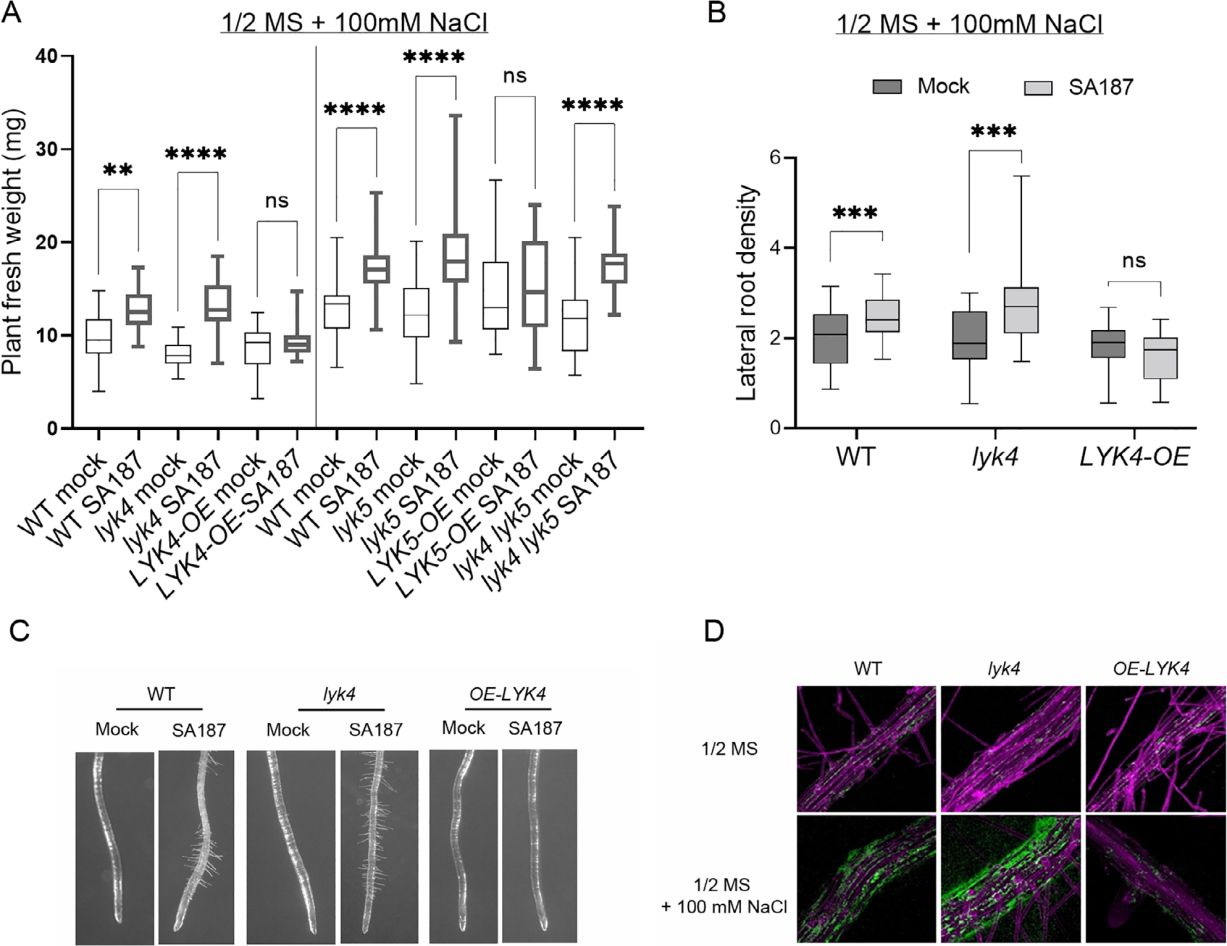
*Enterobacter* sp. SA187‐mediated salt resistance and root phenotypic analysis in *lyk4* and *LYK4* overexpressing line. A. SA187‐induced salt resistance was evaluated by measuring the total plant fresh weight of mock or SA187‐inoculated plantlets after growth for 14 days on 1/2 MS + 100 mM NaCl in WT, *lyk4*, *LYK4‐OE*, *lyk5*, *LYK5‐OE* and *lyk4 lyk5* backgrounds. Since the experiments with the listed genetic backgrounds showed in the graph were not run all together, the vertical black line was used to separate the two different sets of experiments for which the corresponding WT values are reported. Total plant fresh weight of mock or SA187‐inoculated plantlets in WT, *lyk4* and *LYK4‐OE* backgrounds were run in three independent experiments. 25 ≤ no. of plants ≥ 36. Total plant fresh weight of mock or SA187‐inoculated plantlets in WT, *lyk5* and *LYK5‐OE* and *lyk4 lyk5* backgrounds were run in three independent experiments. Number of plants = 33. Statistical analysis was performed by applying the two‐way ANOVA with Bonferroni's test. **P ≤ 0.01; ****P ≤ 0.0001; ns: statistically non‐significant. B. Root lateral density was calculated as the ratio between the no. of secondary roots *per* the primary root length (reported in Fig. S7) measured at 8 days after transferring mock or SA187‐inoculated 5 days old plantlets on plates containing ½ MS + 100 mM NaCl. C. Microscopic observation of root hair morphology of mock or SA187‐inoculated WT, *lyk4* and *LYK4‐OE* plantlets after growth for 13 days on ½ MS + 100 mM NaCl. D. Root colonization pattern of SA187‐*GFP* on WT, *lyk4* and *LYK4‐OE* seedlings grown for 5 days on ½ MS without (upper panel) or with 100 mM NaCl (lower panel).

To identify the PRRs involved in the signalling of SA187‐mediated salt resistance, we tested whether plantlets impaired in well‐described MAMP receptors like *fls2*, *efr1*, *cerk1*, *lyk4* and *lyk5*, show enhanced salt tolerance when treated with SA187. Plantlets of SA187‐treated *fls2*, *efr1* and *cerk1* mutants, which are insensitive to flagellin, elongation factor EF‐Tu and fungal chitin/bacterial peptidoglycan, respectively, behaved like WT under non‐salt conditions (Fig. 3A and B) or upon salt treatment (Supporting Information Table S1 and Fig. S3C and D) suggesting that they do not play a major role in this interaction. Interestingly, salt tolerance of Arabidopsis was enhanced in mutants impaired in *LYK4* (beneficial index: +64 ± 18%, average ± standard deviation) but not in its closest paralogue *LYK5* (+45 ± 6%) when compared with WT (+35 ± 8%) (Fig. 3A and Table S1). The double mutant *lyk4 lyk5* retained the *lyk4* phenotype (+51 ± 7%). *LYK4* and *LYK5* overexpressing lines (OE) were both compromised for SA187‐induced salt tolerance (+12 ± 10% in *LYK4‐OE* and +6 ± 2% in *LYK5‐OE*) (Fig. 3A and Table S1). Importantly, SA187 did not affect growth in WT, *lyk4*, *lyk5*, *lyk4*, *lyk5* as well as *LYK4‐OE* and *LYK5‐OE* under control non‐salt conditions (Supporting Information Fig. S4A–D). Overall, these results suggest that among MAMP receptors, the chitin receptor components *LYK4* and to a lesser extent *LYK5*, play a role in the beneficial interaction between Arabidopsis and SA187.

### SA187 affected plant growth and reshaped root architecture in plants affected in *LYK4* gene

SA187–Arabidopsis interaction was analysed in more detail in *LYK4* affected backgrounds. Both the shoot and root systems in SA187‐colonized *lyk4* plants were significantly more developed than uncolonized plants in presence of 100 mM NaCl, but this beneficial induced by SA187 was completely abrogated in *LYK4‐OE* (Figs 3A and S5A and B).

SA187 colonization also induced major changes in root architecture. Under non‐salt conditions, SA187 induced a higher root density in the WT and *lyk4*, but not in the *OE* line. In the WT and *LYK4‐OE*, this effect was due to an increase in the number of secondary roots, while in *lyk4*, SA187 caused a slight decrease in the primary root length but did not affect the lateral root number (Supporting Information Fig. S6A–C). Under salt stress conditions, in SA187‐colonized *lyk4* plants, lateral root number and density was significantly increased compared to WT (Fig. 3B). In *LYK4‐OE* plants, the enhanced lateral root number and density, which was induced by SA187 in WT plants, was completely abrogated (Fig. S7A and B). Microscopic observation of root architecture highlighted that SA187‐induced root hair formation was enhanced in *lyk4* but completely compromised in *LYK4‐OE* plants, indicating that *LYK4* suppresses SA187‐induced modification of root hair growth (Fig. 3C).

### *LYK4* regulates root colonization by SA187 under salt stress

The engineered strain SA187‐GFP which constitutively expresses the green fluorescent protein (GFP; de Zélicourt *et al*., 2018) was exploited to evaluate the bacterium colonization ability in the different genetic backgrounds. Confocal laser scanning microscopy analysis of plantlets colonized by SA187‐GFP confirmed that under non‐salt conditions, the bacterium colonizes the mature root system but with a preference of the root tip region (de Zélicourt *et al*., 2018) in WT, *lyk4* and *LYK4‐OE* lines (Fig. 3D). When roots were transferred to salt containing medium (100 mM NaCl), colonization was induced in the mature root but preferentially in the newly formed region of the primary root. In comparison to WT, under salt stress conditions, *lyk4* mutant plants showed enhanced root colonization by SA187, whereas root colonization is hampered in *LYK4‐OE* during the early time points of the interaction (Fig. 3D). Re‐isolation experiments from SA187‐treated roots are in agreement with the microscopy data, showing that the bacterium colonization is enhanced in WT under salt stress. Colonization in *lyk4* mutant is significantly higher under both assayed conditions compared to the WT, while *LYK4‐OE* showed lower colonization levels that are not altered on control condition or under salt stress (Supporting Information Fig. S8). These results show that *LYK4* plays an important role in the regulation of SA187 colonization.

### Role of *LYK4* in SA187‐induced reprogramming of global gene expression under control conditions

To uncover the role of *LYK4* in the SA187–Arabidopsis interaction, RNA‐Seq analysis was performed comparing the transcriptomes of non‐colonized (mock) versus SA187‐colonized plants in WT, *lyk4* and *LYK4‐OE* under non‐salt conditions and under salt stress. We first wondered to which extent the SA187 regulated genes depend on the *LYK4* receptor under non‐salt conditions (1/2 MS). Under resting conditions, 1912 genes were differentially expressed (DEGs) in the comparison between non‐colonized and SA187‐colonized WT, *lyk4* and *LYK4‐OE* plants. Using hierarchical clustering, the transcriptome was organized in 20 groups (Supporting Information Fig. S9A and B). Each cluster was analysed for gene ontology enrichment (GO) using DAVID database (Database for Annotation, Visualization and Integrated Discovery; Huang *et al*., 2009; Fig. S10 and File S1).

Cluster 11 and 14 were among the largest ones, consisting of 375 (19.6%) and 186 (9.7%) DEGs, respectively, which were similarly induced in WT, *lyk4* and *LYK4‐*OE upon colonization with SA187 and hence independent of *LYK4* (Supporting Information Fig. S10A and B). In cluster 11, GO terms were affiliated mainly to defence, SA response and systemic acquired resistance (Supporting Information Fig. S10A). In cluster 14, GO terms referred mainly to oxido‐reduction processes, phenylpropanoid biosynthesis, lipid transport, suberin biosynthesis, cell wall organization, and stress response (Supporting Information Fig. S10B). These results indicate that these biological processes are not influenced by *LYK4*.

Cluster 3 encompassed 153 DEGs (8%) that were more strongly induced by SA187 in *lyk4* plants compared to WT plants (Fig. S10C) and were mainly enriched for GO terms of defence response, signalling transduction pathway and chitin response. In the GO term ‘protein phosphorylation’, there is *BAK1*‐*interacting receptor*‐*like kinase 1* (*BIR1*), that encodes a *BAK1*‐interacting receptor‐like kinase that negatively regulates multiple plant resistance signalling pathways (van der Burgh *et al*., 2019) and several members of the CRK receptors (8, 11, 12, 19, 39). The GO term ‘signal trasduction’ included members of the receptor‐like protein (RLP) family (*RLP12*, *21*, *36*).

In cluster 15, 138 DEGs (7.2%) were more repressed in SA187‐colonized *lyk4* and *LYK4*‐*OE* plants than in WT (Supporting Information Fig. S9B). These gene were related to the GO terms oxido‐reduction, lignin biosynthesis, response to stress and water deprivation (Supporting Information Fig. S10D).

Cluster 4 enumerated 149 DEGs (7.8%) that were strongly induced in *LYK4*‐*OE* (Supporting Information Fig. S9B). These genes are mainly related to defence responses (Supporting Information Fig. S10E).

Cluster 10 included 94 (4.9%) DEGs that were strongly repressed in SA187‐treated *LYK4*‐*OE* line compared to WT and *lyk4* (Supporting Information Fig. S9B). The GO term associated with this cluster is starch catabolism (Supporting Information Fig. S10F).

These data suggest that in response to colonization by SA187 under non‐salt conditions, *LYK4* plays a role in coordinating specific defence responses to bacteria, fungi and metabolic activities related to energy storage.

### Transcriptomic overview of the role of *LYK4* in SA187‐induced salt tolerance

**Fig. 4:**
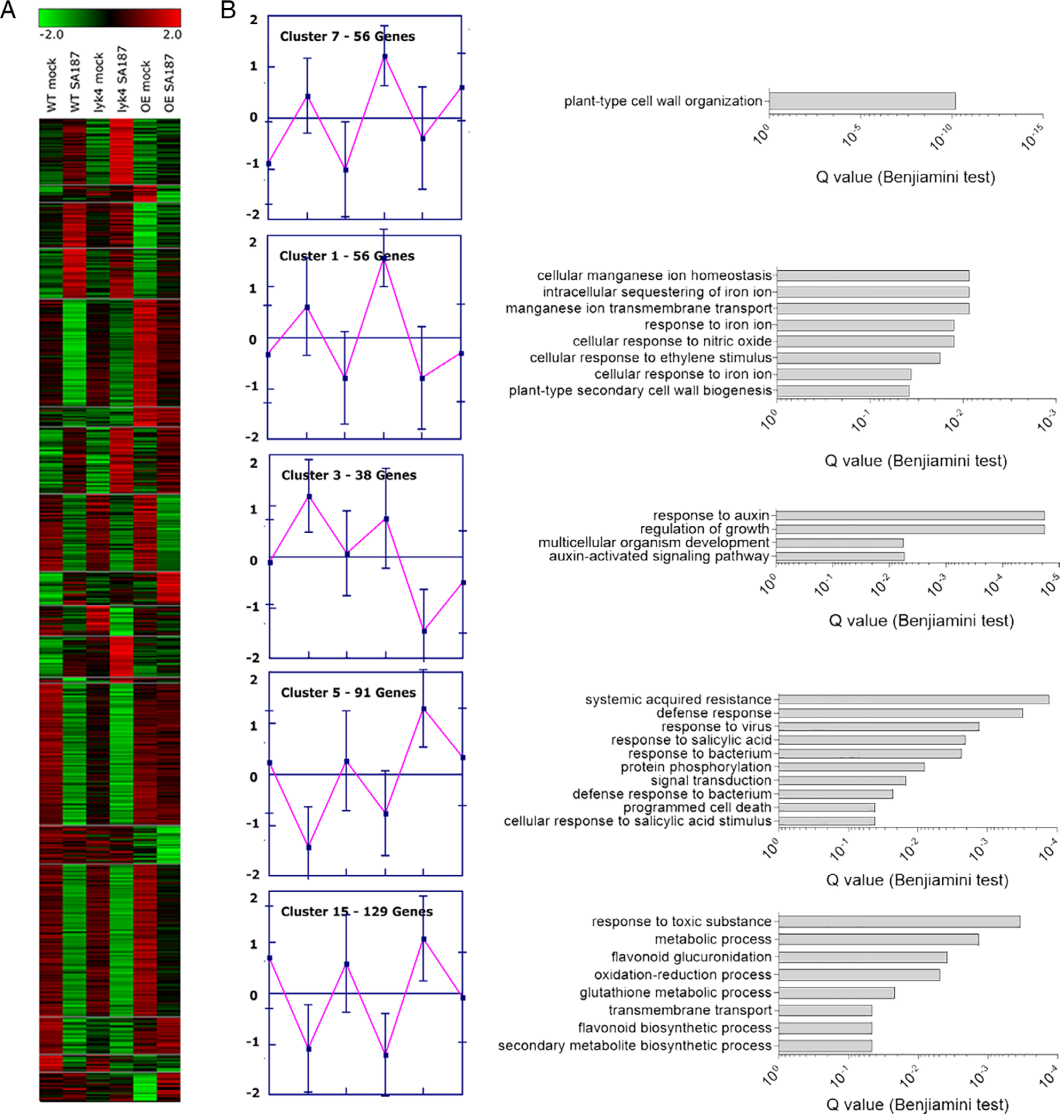
Transcriptome analysis of SA187‐induced salt tolerance in WT, *lyk4* and *LYK4‐OE*. A. Hierarchical cluster of differentially regulated genes in WT, *lyk4* and *LYK4‐OE* under salt stress (1/2 MS + 100 mM NaCl). B. The main clusters of genes that show a differential regulation in *lyk4* and *LYK4‐OE* compared to WT plantlets are shown with centroid expression graphs and the Gene Ontology (GO) enriched terms.

Under salt stress, 819 DEGs were identified in comparing SA187 colonization of WT, *lyk4* and *LYK4*‐*OE*, organized by hierarchical clustering into 18 groups (Figs 4A and S11) and analysed for GO enrichment (File S2 and Fig. 4B). A role of *LYK4* on the SA187‐induced salt stress transcriptome was found in clusters 1, 3, 5, 7, 11 and 15.

Cluster 7 harboured 56 DEGs (6.8%) that were induced by SA187 more in *lyk4* than in WT and *LYK4*‐*OE* plants (Fig. 4B). These genes were involved in cell wall organization and accounted for proteins involved in root remodelling like the proline‐rich extensin proteins (*EXT6*, *8*, *10*, *18*), expansins (*A20*, *B3*) and root hair‐specific protein like *RHS18*. A similar expression pattern is shared by cluster 1, accounting for 56 genes (6.8%) belonging to the GO related to iron and manganese homeostasis, response to ethylene and to cell wall biosynthesis and architecture (Fig. 4B).

In cluster 11 (34 genes, 4.1%), the DEGs were up‐regulated exclusively in SA187‐colonized *lyk4* (Fig. S11): three of these genes are members of the cytochrome P450 family (*CYP705AP12*, *CYP706AP7*, *CYP71BP22*) involved in the biosynthesis of steroids, stilbene, coumarine and lignin (Ehlting *et al*., 2008). Analysis of cluster 11 by STRING showed a network of genes involved in cell wall metabolic process (Supporting Information Fig. S12).

Cluster 3 encoded for 38 DEGs (4.6%) that were repressed in SA187‐treated *LYK4*‐*OE* (Fig. 4B). GO annotation showed that these genes play a role in auxin signalling, including polar transport (like *IAA1*) and auxin‐mediated plant organ development and growth (as, for instance, *SAUR*‐like auxin‐responsive proteins 21–24). Importantly, these genes were already regulated by *LYK4* alone, as *LYK4‐OE* plants show strong down‐regulation of this cluster in the absence of SA187 (Fig. 4B).

The insensitivity of *LYK4‐OE* plants to the beneficial effect of SA187 to salt stress might be related to the higher expression of DEGs in cluster 5, encoding 91 genes (11.1%) with the GO terms response to SA and defence response (Fig. 4B). These genes include *CRK6*, which is induced by *NPR1* in response to SA (Wrzaczek *et al*., 2010), *WRKY70*, a well‐known activator of SA‐dependent defence responses (Zhou *et al*., 2018) and also *LYK5*.

**Fig. 5:**
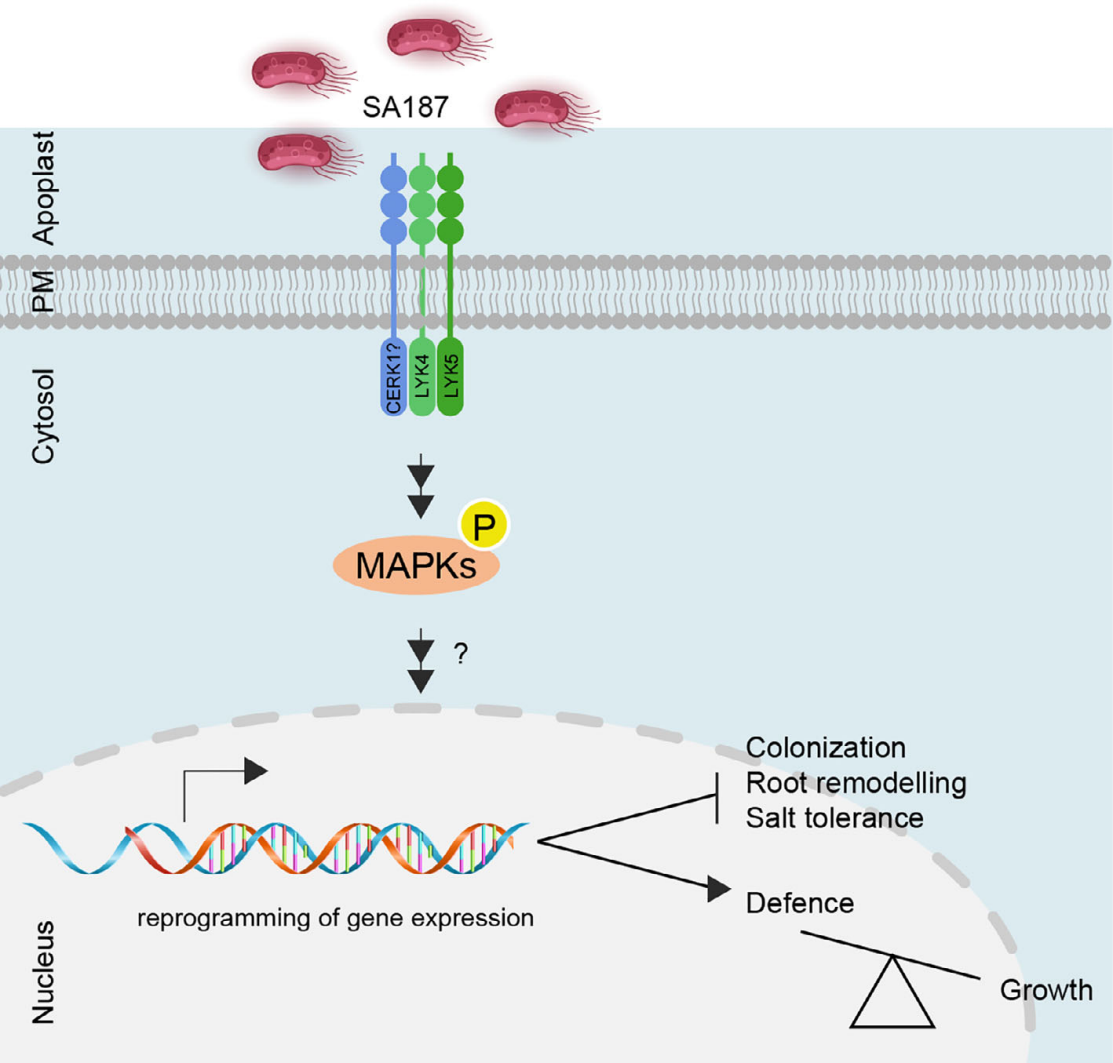
Proposed model explaining the role played by *LYK4* in SA187‐mediating salt stress tolerance. Schematic representation of the main findings presented in the present study on phenotypic, microscopy and transcriptomic analysis summarizing the role of *LYK4* in SA187‐Arabidopsis interaction under salt stress. The arrow indicates a direct positive effect, suggesting that *LYK4* favours defence responses over growth. The other line indicates that *LYK4* plays an inhibitory role on the indicated SA187‐promoted processes.

In cluster 15, 129 DEGs (15.7%) were associated with response to toxic substances, and flavonoid synthesis and glucoronidation. These genes were suppressed by SA187 colonization in WT and *lyk4*, but to a lesser extent in *LYK4‐OE* plants (Fig. 4B). These genes are involved in flavonoid biosynthesis, a large class of secondary metabolites implicated in pigmentation, redox and UV protection, but which also play a role in plant–microbe interaction (Schäffner, 2016). A role of *LYK4* in metabolic process is provided by cluster 13. Genes of this cluster are not regulated by SA187 but are strongly upregulated in *LYK4*‐*OE* (Supporting Information Fig. S11) and encode genes for rRNA processing. Overall, the transcriptome analysis suggested that *LYK4* interferes with SA187‐triggered root morphogenetic programme, potentially involved in salt resistance (Fig. 5).

### *LYK4* plays a role in mediating bacterially produced KMBA in SA187‐induced plant salt tolerance

The ability of SA187 to improve plant tolerance to salinity is mediated by the manipulation of ethylene signalling. α‐Keto‐γ‐(methylthio) butyric acid (KMBA), an ethylene precursor (Eckert *et al*., 2014), is produced by SA187 through the methionine salvage pathway and, via its conversion to ethylene *in planta*, plays a key role in the beneficial activity of SA187 (de Zélicourt *et al*., 2018). To test whether *LYK4* was involved in mediating the ethylene‐mediated induction of Arabidopsis salt tolerance, the effects of the ethylene precursors KMBA and 1‐aminocyclopropane‐1‐carboxylic acid (ACC) were tested on WT, *lyk4* and *LYK4*‐*OE* plants. *lyk4* plants showed an enhanced response to KMBA and ACC when compared to WT, increasing salt tolerance (Supporting Information Fig. S13 and Table S2), while in contrast, *LYK4‐OE* plants compromised KMBA‐induced salt tolerance (Supporting Information Fig. S12 and Table S2). Similar results were observed in shoot and root fresh weight (Supporting Information Fig. S12B and C).

Taken together, these results show that *LYK4* is involved in regulating the bacterial‐derived metabolite ethylene signalling pathway during the beneficial interaction of SA187 with Arabidopsis (Fig. 5).

## Discussion

In soil, plant roots interact simultaneously with a plethora of commensal, beneficial and pathogenic microorganisms that expose various types of MAMPs (Yu *et al*., 2019). Through still largely unclear mechanisms, the plant immune system can discriminate between harmful and beneficial interactions, taking advantage of the microbe‐provided ecological services that can support its fitness (Trdá *et al*., 2015; Plett and Martin, 2018). Increasing the knowledge of the plant immune regulation under abiotic stress will foster the engineering of the plant microbiome (Saikkonen *et al*., 2020) to face adverse environmental conditions, like increasing soil salinization (Shahzad *et al*., 2021).

The dosage of SA187 plays a role in inducing immune responses and this can have implications for the dynamic interplay of the plant with microbes in the rhizosphere, a hotspot for microbial diversity and density. The rhizosphere inhabiting population size is estimated to encompass 10^8^–10^9^ bacteria per gram of plant root fresh weight (Reinhold‐Hurek *et al*., 2015). How plants evolved detection systems able to manage simultaneously such large and diverse interactions is still unknown. For SA187, the observed threshold for plant immune activation corresponded to 7 × 10^6^ CFU ml^−1^. This result is in agreement with previous reports where root exposure to similar bacterial concentrations failed to induce an immune response in Arabidopsis roots (Millet *et al*., 2010; Lakshmanan *et al*., 2012). While rhizobacteria express flagellins that are poorly recognized by the plant immune system (Lopez‐Gomez *et al*., 2012), and the grapevine *FLS2* receptor discriminates between flagellin molecules produced by the beneficial strain *P*. *phytofirmans* and by pathogens (Trdá *et al*., 2014), SA187 immunogenic flagellin peptides triggered canonical immune responses in Arabidopsis. These results are surprising as pathogenic and beneficial microbes alike have been shown to actively suppress the immune system in Arabidopsis (Millet *et al*., 2010). However, given the fact that SA187 can colonize the rhizoplane of various plant species and also establish in the endosphere (de Zélicourt *et al*., 2018), SA187 must have evolved alternative strategies to avoid or deal with the plant immune system. Although SA187 lacks the components for a bacterial T3SS (Andrés‐Barrao *et al*., 2017), which is necessary for MTI suppression by phytopathogenic bacteria (Alfano and Collmer, 2004), SA187 is endowed with T2SS, that mediates extracellular secretion of multimeric proteins, and with the poorly characterized T6SS, that mediates translocation of effector proteins targeting the bacterial cell wall and membrane, supporting a role in bacterial competition with other microorganisms (Russell *et al*., 2014). Both secretion systems were described in pathogenic as well as in non‐pathogenic bacteria (Korotkov *et al*., 2012; Shyntum *et al*., 2014). Therefore, SA187 might interfere with host immunity by T2SS or T6SS systems to suppress MTI.

To identify the putative membrane‐associated receptor complex that mediates SA187‐induced plant salt tolerance, a number of PRR mutants for known MAMP signalling pathways were tested in the phenotypic assay of SA187‐induced salt tolerance in Arabidopsis (de Zélicourt *et al*., 2018). A similar salt promotion effect as in WT was observed in knock‐out plants for *FLS2*, that recognized almost all SA187‐encoded flagellin peptides, for *fls2 efr1*, impaired also in recognition of the bacterial elongation factor Tu (Zipfel *et al*., 2006), for *cerk1*, acting as a functional hub for immunity and abiotic stress perception (Espinoza *et al*., 2017). Therefore, none of these PRRs mediates SA187‐triggered beneficial effect on Arabidopsis salt tolerance. Compared to WT plants, SA187 significantly enhanced salt resistance in *lyk4* and to a lesser degree in *lyk5* mutant and importantly, the SA187‐induction of salt tolerance in Arabidopsis was completely compromised in *LYK4* or *LYK5* overexpressing plants. The present work widens the knowledge on LysM receptors, showing that *LYK4*, previously supposed to play an ancillary role in chitin signalling and recently demonstrated to be a component of the chitin receptor (Xue *et al*., 2019), has a key role in beneficial plant–bacterial interaction. In *lyk4* mutant plants, SA187‐induced changes in root architecture were mainly due to a higher lateral root density: this phenotype may contribute to the enhanced salt tolerance in SA187‐colonized plants. Moreover, SA187‐colonized *lyk4* roots show enhanced proliferation of root hairs whereas *LYK4‐OE* plants are basically devoid of root hairs. Given that root hairs are essential components for the efficient acquisition of nutrients and water (Tanaka *et al*., 2014), root hair proliferation may affect plant fitness under abiotic stress conditions. Indeed, root architecture is a key trait for plant adaptation to abiotic stresses conditions like salinity (Koevoets *et al*., 2016) and beneficial bacteria contribute to remodel root development in a strain‐specific manner (Garrido‐Oter *et al*., 2018). Microscopic inspection confirmed a role of *LYK4* in SA187‐induced root hair growth and development.

To analyse the interaction of SA187 with Arabidopsis at the molecular level, the transcriptome of SA187‐colonized and non‐colonized Arabidopsis plantlets grown under salt and non‐stress conditions was compared. Despite undetectable morphological changes in SA187‐colonized *lyk4* plants, profound changes in the transcriptome landscape were already observed under non‐salt conditions. As shown by the oppositely altered expression levels of genes related to plant defence in *lyk4* and *LYK4‐OE* plants, *LYK4* seems to be involved in regulating some aspects of Arabidopsis immunity during SA187 interaction. In particular, the differential expression for several cysteine‐rich receptor like kinases (CRKs) and RLPs in SA187‐colonized *lyk4* and *LYK4‐OE* plants provide hints for the identification of PRRs involved in SA187 perception and interaction. CRKs belong to a large gene family of receptors, but several play important roles in plant defence and immunity (Wrzaczek *et al*., 2010). Among these, differential regulation in *LYK4* mutants was found for *CRK8*, which may act as ROS sensors (Bassal *et al*., 2020) as well as *CRK9*, which plays a redundant function with *CRK4* and *CRK5* in ABA signalling (Lu *et al*., 2016). The differential regulation of several regulators of MAPK signalling, such as *PUB23* and *PUB24* (Trujillo *et al*., 2008), and MAPK targets, such as *WRKY48* (Xing *et al*., 2008) and *WRKY53* (Wan *et al*., 2004), substantiate that *LYK4* plays an important role in regulating defence reactions during SA187–Arabidopsis interaction. Interestingly, several differentially expressed genes in the interaction of SA187 with *lyk4* or *LYK4*‐*OE* link *LYK4* to the regulation of stress tolerance. Among these genes, several are related to ABA, water deprivation, but also to metabolic processes like starch metabolism.

Under salt stress, several sets of genes showed differential regulation when compared to non‐salt conditions, indicating that *LYK4* is involved in processes that are not only of importance in plant–microbe interaction. Under salt stress, SA187‐colonized *lyk4* plants showed upregulation of genes involved in the biosynthesis and remodelling of the plant cell wall and root hair elongation, consistently with the observed SA187‐induced changes in root architecture in the mutant line. The *LYK4*‐dependent DEGs include *LAC4*, a peroxidase that contribute to the deposition of lignin in secondary cell wall (Chou *et al*., 2018), FLA12 that ensures the integrity and elasticity of the plant cell wall matrix (Johnson *et al*., 2011), *EXT13* and *EXT8*, extensin proteins associated with root hair development that structures a distinctive network in root hair cell walls through cross‐links via peroxidases (Velasquez *et al*., 2011). For SA187‐induced root hair phenotype, specific genes were identified such as *RHS18*, a family of proteins with an elusive molecular function that appears important for cell wall biogenesis and remodelling (Shibata and Sugimoto, 2019).

Interestingly, the genes of cluster 3 are mainly related to auxin signalling and cell growth, showing reduced expression in *OE‐LYK4* compared to WT and *lyk4* upon colonization by the beneficial bacterium. GO analysis of these genes identified several members of the family of small auxin upregulated RNA *SAUR* (21–24) genes that regulate auxin‐mediated cell elongation and expansion (Spartz *et al*., 2012) as well as *IAA* genes. The gene list includes cell wall modifying enzymes, consistent with the role played by auxin in root morphogenetic processes, including in particular lateral root emergence. Indeed, auxin‐induced acidification induces expression of expansins, such as *EXP11*, that loosen the cell wall at cellulose‐xyloglucan junctions and promotes XTH proteins that aid in loosening of cell walls, such as *XTH4*. These findings are of particular interest, considering that both ethylene and auxin synergistically regulate root hair growth and elongation (Shibata and Sugimoto, 2019). Blocking ethylene perception or inhibiting ethylene synthesis reduces the number of root hairs and similarly auxin promotes root hair formation (Qin and Huang, 2018). These data suggest that *LYK4* might play a role in mediating SA187 signals to alter cell growth and development of the root system.

Together with a reduced expression of auxin responsive genes and cell wall remodelling enzymes, the enhanced expression of SA‐responsive defence genes and of flavonoids could be the cause for the loss of SA187‐triggered salt tolerance in *LYK4‐OE* plants. Higher SA levels show inhibitory effect on auxin uptake, sensitivity and signalling, thus prioritizing defence over growth, whereas, in the absence of biotic challenges, auxin attenuates SA‐mediated defences, boosting growth (Naseem *et al*., 2015). Although *LYK4* does not seem to affect the early events in plant immune responses, the higher expression of flavonoid‐encoding and SA‐dependent genes could explain the reduced colonization ability of SA187 on *LYK4‐OE* roots. Indeed, root system hypercolonization by the biocontrol endophyte Fo47 was observed only in MTI‐compromised tomato plants (de Lamo *et al*., 2021).

In an effort to identify the microbial determinants improving SA187 enhanced beneficial response in *lyk4* plants, we assayed the bacterial metabolite KMBA that was shown to induce salt tolerance in Arabidopsis (de Zélicourt *et al*., 2018). Since KMBA is a metabolite that can be converted into ethylene *in planta*, we also tested ACC, the direct precursor of ethylene. As seen for SA187, both KMBA and ACC could enhance salt stress tolerance in *lyk4* mutants to higher levels than in WT plants. These effects were clearly compromised in *LYK4‐OE* plants, indicating that *LYK4* is involved in SA187‐mediated ethylene signalling in Arabidopsis. At present, it is not clear whether this occurs by a direct or indirect interaction of KMBA or ACC with *LYK4* or other components of the ethylene signalling cascade.

In conclusion, we report a new role for *LYK4* in regulating the beneficial plant‐microbe interaction of SA187 with Arabidopsis, suggesting that it acts as a negative regulator of the bacteria‐triggered salt resistance (Fig. 5). Our findings suggest a functional trade‐off between defence and root developmental programmes to ameliorate effects on the plant fitness under abiotic stress conditions, with crucial biotechnological implications to exploit beneficial microbes for ensuring crop yield and enhancing plant resilience during environmental stress conditions.

## Experimental procedures

### Bacteria and plant material used in the present study

*Enterobacter* sp. SA187 was previously isolated from root nodules of the leguminous pioneer plant *Indigofera argentea* in the Jizan region of Saudi Arabia (Andrés‐Barrao *et al*., 2017). Arabidopsis Col0 was used for the described assays and the following mutants were used in the present study: *fls2* (SALK_141272 and SALK_093905), *fls2 efr1* (SAIL_691C4; SALK_044334C), *cerk1‐2* (GABI‐KAT 096F09), *lyk4* (WiscDsLox297300_01C), *lyk5‐2* (SALK_131911C). The mutant line *lyk4 lyk5* and the *LYK4* and *LYK5* overexpressing lines were published previously (Liang *et al*., 2013). All plants were grown in long day conditions in growth chambers (Percival; 16 h light/8 h dark, 22°C).

### Bacterial growth conditions

SA187 was routinely grown on LB medium. an overnight pre‐culture was prepared by inoculating the bacterium directly from the glycerol stock in 20 ml LB for grow o/n at 30°C under shaking conditions. The next morning, the bacterium was re‐inoculated in fresh LB liquid medium (generally 250 μl in 25 ml of medium) and the growth of the strain was followed until the exponential phase, typically 0.6–0.8 OD. Appropriate dilutions were prepared, as described in the next paragraphs.

### Arabidopsis growth conditions and root architecture analysis

Prior to every experiment, *A*. *thaliana* seeds were surface sterilized for 10 min in 70% ethanol + 0.05% sodium dodecyl sulfate on a shaker, washed two times in 96% ethanol and allowed to dry. For the MAPK activation assays and for the evaluation of the SA187 immunogenic response, Arabidopsis seedlings were grown in liquid 1/2 MS (Murashige and Skoog basal salts, Sigma, 0.5% MES, pH 5.4) supplemented with 1% sucrose. Seeds were vernalized for 48 h in the dark at 4°C and then grown for 11 days at 22°C under long‐day conditions. At day 11, the MS medium was carefully removed from the plates, the plantlets were washed carefully and rapidly with 5 ml of fresh 1/2 MS without sucrose to remove any traces of sugar and finally 5 ml of fresh 1/2 MS (no sucrose) was added in each plate. The plantlets were placed again in the growth cabinet and the day after they were treated with liquid MS alone (mock) or supplemented with the flagellin peptides (1 μM or 100 nM) or with a bacterial dilution (OD = 0.5, corresponding to 2 × 10^8^ CFUs ml^−1^ and OD = 0.1, corresponding to 2 × 10^7^ CFUs ml^−1^ and OD = 0.01, corresponding to 2 × 10^6^ CFUs ml^−1^) at the given concentrations. The flg22 peptide was bought from GeneCust Europe; the SA187‐flg22 peptides were bought from Genecust (2 mg with >85% purity). P1 and P4 were resuspended in sterile ultrapure H_2_O, while P2 and P3 were resuspended in 70% dimethylsulphoxide.

Samples were collected according to a time course (15–30–60 min), rapidly frozen in liquid nitrogen and stored at −80°C until further use.

For growth inhibition assays, seedlings were grown in solid MS plates, transferred to liquid MS with or without the flagellin peptides at day 5, and weighted 7 days after treatments.

For the salt stress tolerance assays, sterilized seeds were sown on ½ MS plates containing or not SA187 (2 × 10^5^ CFUs ml^−1^) that was supplemented as previously described. The seeds were stratified for 2 days at 4°C in the dark and then the plates were placed vertically to grow for 5 days. Five‐day‐old seedlings were transferred onto ½ MS plates with or without 100 mM NaCl (Sigma) and this was considered T0 of the assay. Primary root length was measured at day 8 after transfer using ImageJ software on scanned images of the plates. The number of lateral roots was evaluated as detectable number of lateral roots observed under a stereo microscope. Fresh weight of shoots and roots was measured 14 days after transfer of seedlings. The beneficial index, that is the salt promotion ability exerted by SA187, was calculated as the ratio between fresh weight ‐SA187 and ‐mock inoculated seedlings and was expressed in percentage in the same genetic background. Operatively, it was calculated on the average value of the fresh weight (SA187 and mock‐treated plants) obtained in each biological replicate. Thus, the values of the beneficial index showed in Table S1 correspond to the average ± standard deviation of the beneficial index calculated in each biological replicate. The statistical analysis was performed by applying the Mann and Whitney test. The beneficial index values of each replicate are indicated in Table S1. The beneficial index was used as a tool to compare SA187‐salt improving performances in the different mutant backgrounds used in the present study.

### GUS staining

To evaluate the immune response elicitation and suppression the reporter line *pCYP71A12*::*GUS* (Denoux *et al*., 2008) was used according to Millet *et al*. (2010). For the elicitation assay, seeds were germinated on ½ MS basal salts, 9% agar, pH 5.8. Overnight cultures of SA187, *P*. *syringae* pv. tomato DC3000 (Pst) or hrcC‐ in King's medium were centrifuged at 2500*g* for 5 min, washed three times and diluted in sterile distilled water to a final OD = 0.1. Ten days after germination, *pCYP71A12*::*GUS* expressing seedlings were placed horizontally and covered for 5 h with a solution of 10 μM Flg22 or heat inactivated bacteria at OD = 0.1. To evaluate the ability of bacteria to suppress the immune response, sterile seeds were germinated on ½ MS basal salts, 9% agar, pH 5.8, supplemented with 100 μl fresh cultures of Pst DC3000, hrcC‐ or SA187 at logarithmic phase (OD ≈ 0.4–0.5) per 50 ml of medium. The seedlings were then placed horizontally and covered with ½ MS basal salts, pH 5.8 with 10 μM Flg22 for 5 h. Then the seedlings were vacuum infiltrated and incubated in staining solution (200 mM sodium phosphate, buffer, pH 6.7, 5 μM potassium ferricyanide (K_3_Fe(CN)_6_), 5 μM potassium ferrocyanide (K_4_Fe(CN)_6_), −0.01% Triton‐X, 50 μg ml^−1^ of 5‐bromo‐4‐chloro‐3‐indolyl‐b‐d‐glucuronic acid (X‐Gluc; Sigma), 50 mM sodium phosphate buffer (pH 7.2)]. The reaction was incubated at 37°C in darkness until signal was developed. Stained seedlings were washed, dehydrated and cleared in 90% acetone, 75%, 50% and 25% ethanol series, mounted on slides with chloral hydrate overnight and observed with Axio Imager 2 (Zeiss) equipped with Plan‐Neofluar 10x/0.45 objective.

### Laser scanning confocal microscopy imaging

For confocal microscopy, we used the GFP genetically integrated the wild‐type SA187, termed SA187‐GFP as in de Zélicourt *et al*. (2018). Five days old seedlings were used to visualize the colonization of SA187‐GFP on roots. Embedding medium was 100 μg ml^−1^ propidium iodide (PI) in distilled water. We used the confocal microscope ZEISS LSM880 with Airyscan with the Plan‐Apochromat 10× (n.a. 0.45) objective lens. For excitation of both GFP and PI, we used the argon‐based laser with power output 6.5. Pinhole was set to 1 A.U. Emission filters were set to 493–584 nm for GFP and 604–718 nm for PI. For Z‐stacking, we used a minimal number of scans (12–20) in order to have at least 10% overlap between channels. We used, the ZEN black edition 3.0 SR for assembly of images and the ZEN blue lite edition 3.0 was used for annotations, image cropping and extraction.

### SA187 re‐isolation from the root system of colonized plantlets

To evaluate the early effects of salt stress on SA187 root colonization efficiency, plates containing WT, *lyk4* and *OE‐LYK4* colonized plantlets were scanned after 3 days of growth on 1/2 MS and 1/2 MS + 100 mM NaCl and primary root length was measured by using ImageJ software. Roots were cut by the use of a sterile blazer and blended in sterile physiological buffer (9 g L^−1^ NaCl) and then aliquots of serial dilutions (10^0^–10^−7^) were plated.

### SA187 flagellin peptides alignments

Five paralogs of the gene coding for flagellin (*fliC*) were identified in the SA187 genome sequence (Andrés‐Barrao *et al*., 2017). The sequence of the five paralog proteins were obtained from the genomic information and aligned to the flg22 peptide by using ClustalW tool available at ExPASy (www.expasy.org) (Artimo *et al*., 2012). The conserved peptide sequence was extracted and similarity tree was constructed using MEGAX software (Kumar *et al*., 2018).

### Protein extraction and Western blot analysis

Total proteins were extracted using a native buffer containing 50 mM Tris–HCl pH 7.5, 150 mM NaCl, 0.1% NP40, 5 mM EGTA, 0.1 mM DTT, 1 complete protease inhibitors (Roche Applied Science), 1 mM NaF, 0.5 mM Na_3_VO_4_, 15 mM β‐glycerophosphate, 15 mM 4‐nitrophenyl phosphate, and protein quantification for normalization was performed using a Bradford assay. Chemicals were purchased from SIGMA unless otherwise stated. Protein samples were separated on 10% acrylamide/bis‐acrylamide (SIGMA) SDS‐PAGE gels and transferred on 0.45‐μm PVDF membranes (GE Healthcare). Blocking was performed in TBST containing 5% BSA, blots were incubated overnight with anti‐pTpY antibody (rabbit anti‐phospho‐p44/42 MAPK (Erk1/2) (Thr202/Tyr204; Cell Signalling), washed in TBST, and incubated with the goat anti‐rabbit horseradish peroxidase (HRP)‐conjugated secondary antibody (SIGMA) diluted to 1/10 000. The chemiluminescent detection of HRP was performed with ECL reagent (GE Healthcare) and an imaging system (GeneGnome; Syngene). After immunoblotting and imaging, the blots were stained with a solution of Coomassie blue for protein visualization: 0.75% Coomassie Brilliant Blue R‐250 (821616, ICN Biomedicals), 20% ethanol, 10% acetic acid.

### RNA extractions

Seedlings were harvested at the indicated times and frozen in liquid nitrogen. Total RNA extractions were performed using the NucleoSpin RNA kit (Macherey‐Nagel) following the manufacturer's recommendations. RNA quantification and quality were measured in a NanoDrop (Thermo Scientific).

### RNA‐Seq and qRT‐PCR analysis

Total RNA was extracted from 5‐day‐old plants either or not inoculated with SA187 and transferred for 14 more days on ½ MS plates with or without 100 mM NaCl using the Nucleospin RNA plant kit (Macherey‐Nagel), including DNaseI treatment and following manufacturer's recommendations.

mRNA libraries were prepared using 1 μg of total RNA using TruSeq Stranded mRNA Library Prep kit (Illumina). Sequencing of the pooled libraries was performed on HiSeq 4000 platform. Paired‐end sequencing of RNA‐Seq samples was performed using Illumina GAIIx with a read length of 100 bp. Reads were quality‐controlled using FASTQC (https://www.bioinformatics.babraham.ac.uk/projects/fastqc/). Trimmomatic was used for trimming of adaptor sequences (Bolger *et al*., 2014). Parameters for read quality filtering were set as follows: minimum length of 36 bp; Mean Phred quality score greater than 30; leading and trailing bases removal with base quality below 3; sliding window of 4:15. TopHat v2.1.1 was used for alignment of short reads to the *A*. *thaliana* genome.

Differentially expressed genes among mock vs SA187‐treated condition were identified using DESeq2 R language‐based tool (Anders and Huber, 2010; Love *et al*., 2014). It calculates the DEGs using a generalized‐linear model and are based on the negative binomial distribution. Benjamini–Hochberg correction was used to correct for sample comparisons (with a false discovery cut‐off < 0.05). Genes were considered as deregulated if fold change was >log2^|0.6|^ and adjusted *p* < 0.05 compared to Mock condition. For the hierarchical clustering and gene family enrichment, Arabidopsis regulated genes were used to generate *K*‐mean clustering using Genesis (Sturn and Quackenbush, 2002) using no. of clusters = 18.24; interaction = 100; random = 50.

RNA‐Seq data set can be retrieved under accession ID GSE157443 at GEO datasets (https://www.ncbi.nlm.nih.gov/gds).

For qPCR analyses, cDNAs were synthesized from 1 μg total RNA using oligo(dT) and SuperScript III reverse transcriptase (Invitrogen) in a 30‐μl total volume. For the synthesis of cDNA, RNAs were heated at 65°C for 10 min, RT mix was added, and the reaction proceeded at 42°C for 1 h. cDNAs were then heated at 75°C for 15 min and stored at 4°C. qPCR reactions were performed in 10 μl final volume with 1 μl RT reaction, 100 nM final concentration of each primer pair, and SYBR FAST Universal qPCR kit (Kapa Biosystems). Primer sequences are listed in Supporting Material S1. ACTIN2 (At3g18780) was used as reference gene. All reactions were performed in a CFX384 Touch™ Real‐Time PCR Detection System (Bio‐Rad) as follows: 95°C for 30 s, ×40 95°C for 5 s, and 60°C for 20 s; and a dissociation step was programmed to validate the PCR products. qPCR was carried out on a CFX384 Touch Real‐Time PCR device (Bio‐Rad) and analysed with CFX Manager Software (Bio‐Rad). qRT‐PCR validation is reported in Fig. S14 and the RNA‐seq values are reported in Table S3. The expression of SA187‐FliC genes was evaluated by qRT‐PCR as previously described by Andrés‐Barrao *et al*. (2017).

### STRING analysis

PPI networks of nutrient‐responsive proteins were generated with STRING (http://string.embl.de) based on known and predicted interactions and displayed in confidence view.

### Statistical analysis

Statistical analysis and graph elaboration was performed by Prism‐GraphPad version 9.2.0.

## Supporting information


**Appendix S1:** Supplementary Information

**File S1** The excel file contains the information about hierarchical clusters and the main GO associated terms for the 1912 DEGs that are differentially expressed among mock and SA187 treated WT, *lyk4* and *OE‐LYK4* under control conditions (1/2 MS).

**File S2** The excel file contains the information about hierarchical clusters and the main GO associated terms for the 819 DEGs that are differentially expressed among mock and SA187 treated WT, *lyk4* and *OE‐LYK4* under salt stress (1/2 MS + 100 mM NaCl).
