# Supplementary material for: The Lys‐motif receptor LYK4 mediates Enterobacter sp. SA187 triggered salt tolerance in Arabidopsis thaliana
Source: Environ Microbiol. 2021 Dec 23;24(1):223–39. doi: 10.1111/1462-2920.15839 (PMC9304150; doi:10.1111/1462-2920.15839)
Supplement: Supplementary file 1 — Appendix S1: Supplementary Information [file EMI-24-223-s001.docx]

**SUPPORTING INFORMATION**

**The Lys-motif receptor *LYK4* mediates *Enterobacter* sp. SA187 triggered salt tolerance in *Arabidopsis thaliana*.**

Eleonora Rolli^1*^, Axel de Zélicourt^1^, Hanin Alzubaidy^2^, Michael Karampelias^2^, Sabiha Parween^2^, Naganand Rayapuram^2^, Baoda Han^2^, Katja Froehlich^2^, Aala A. Abulfaraj^3^, Hanna Alhoraibi^4^, Kiruthiga Mariappan^2^, Cristina Andrés-Barrao^2#^, Jean Colcombet^1&^, Heribert Hirt^2,5&^.

^1^ Université Paris-Saclay, CNRS, INRA, Université Evry, Institute of Plant Sciences Paris-Saclay (IPS2), 91405, Orsay, France

^2^ DARWIN21, Center for Desert Agriculture, King Abdullah University of Science and Technology, Thuwal, Saudi Arabia.

^3^ Department of Biological Sciences, Science and Arts College, Rabigh Campus, King Abdulaziz University, Jeddah, Saudi Arabia.

^4^ Department of Biochemistry, Faculty of Science, King Abdulaziz University, 21551 Jeddah, Saudi Arabia.

^5^Max F. Perutz Laboratories, University of Vienna, Dr. Bohrgass 9, 1030 Vienna, Austria

*present address: Department of Food, Environmental and Nutritional Sciences (DeFENS); University of Milan, via Celoria 2; 20133 Milan, Italy.

# present address: Red Sea Research Center, King Abdullah University of Science and Technology, Thuwal, Saudi Arabia.

& these authors co-supervised equally the work.

Co-corresponding authors:

Jean Colcombet, Institute of Plant Sciences Paris-Saclay (IPS2), 91405, Orsay, France. Phone number: +33 (0)1 69156818; Mail address: jean.colcombet@inrae.fr

Heribert Hirt, DARWIN21, Center for Desert Agriculture, King Abdullah University of Science and Technology, Thuwal, Saudi Arabia. Phone number: 00966 (0)544701080; Fax Number: 00966 (0)12 8082959; Mail address: heribert.hirt@kaust.edu.sa.

**SUPPORTING FIGURES**

**
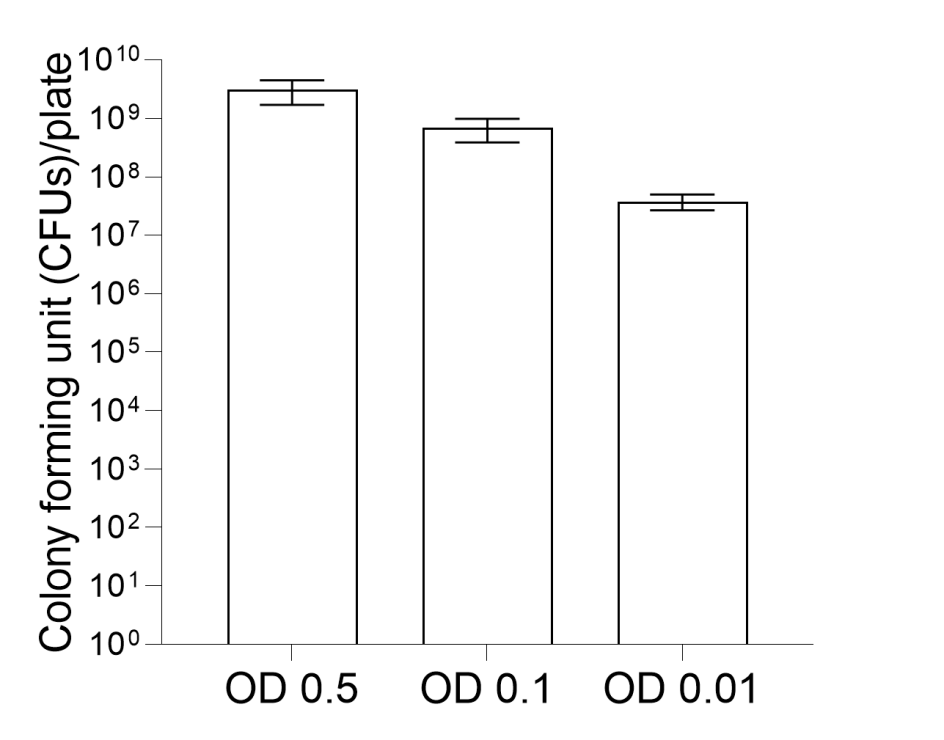
**

**SUPPORTING FIGURE 1. SA187 bacterial density used to induce plant immune activation in 11 days old Arabidopsis plantlets.**

The graph reported the correspondence between the optical density (OD) and the colony forming units (CFUs) for the SA187 bacterial suspensions used to test the ability of SA187 to induce the plant immune response in 11 days old Arabidopsis plantlets. The plantlets were grown in petri dishes with 5ml of ½ MS liquid medium. For the treatment with SA187 bacterial at OD=0.5, plantlets were exposed to a total bacterial load of (3.11±1.99) x 10^9^ CFUs/plate, corresponding to 6.22 x 10^8^ CFUs/mL. For the treatment with SA187 bacterial at OD=0.1, plantlets were exposed to a total bacterial load of (6.86±5.22) x 10^8^ CFUs/plate, corresponding to 1.4x 10^8^ CFUs/mL. For the treatment with SA187 bacterial at OD=0.01, plantlets were exposed to a total bacterial load of (3.81±1.98) x 10^7^ CFUs/plate, corresponding to 7.6x 10^6^ CFUs/mL.


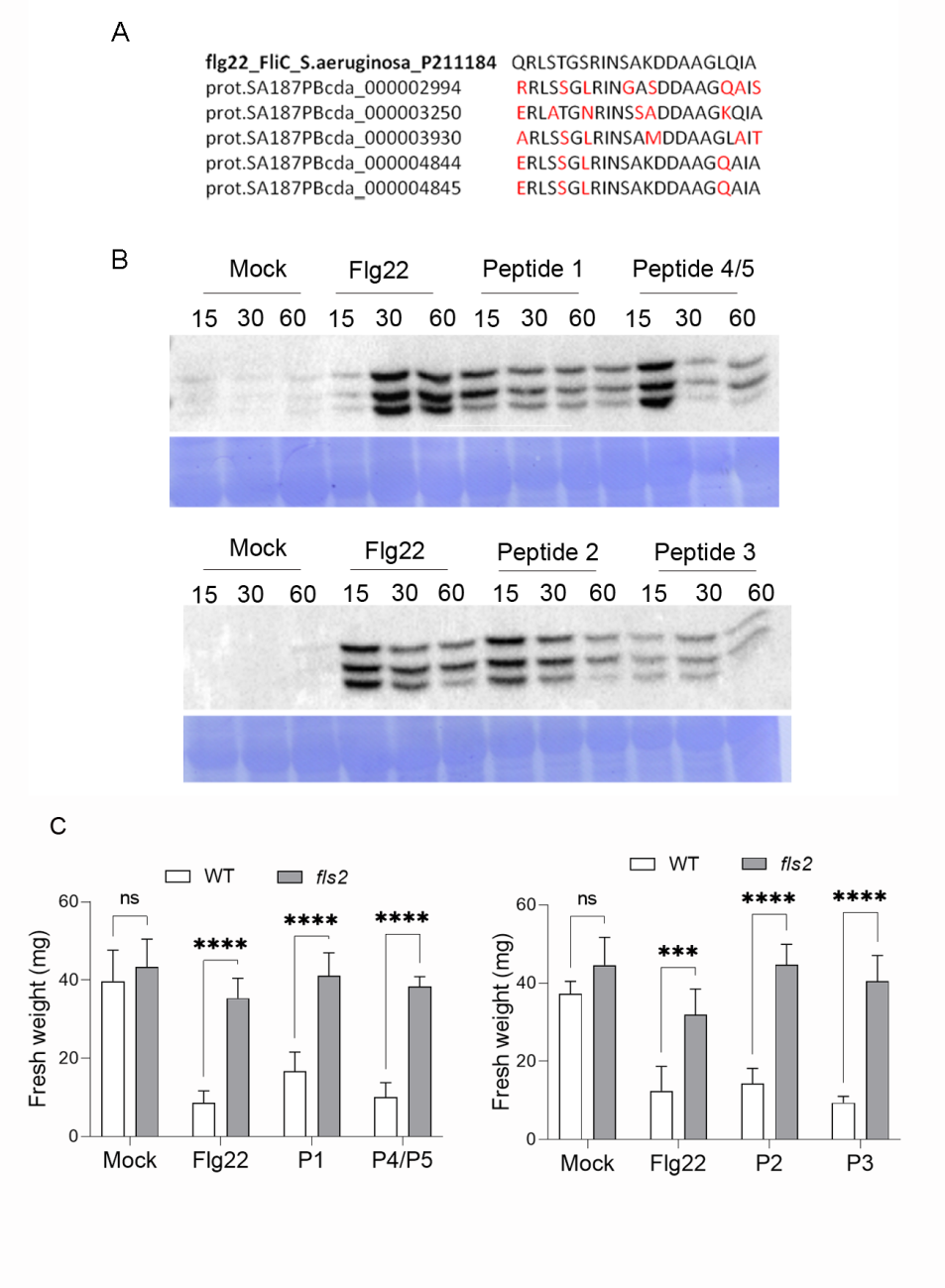


**SUPPORTING FIGURE 2. SA187 encoded flagellins are recognized as MAMPs by the plant immune system.**

**(A)** Alignments of the N-terminal immunogenic sequence of 22aa flagellin encoded by *P. aeruginosa* compared to SA187-Flg encoded peptides. In the first column the gene code identification of the flagellin genes in SA187 genome are reported; in the second column, the alignment is reported for the 22 aminoacids peptides. Changes in amino acid composition in SA187-Flg peptides are highlighted in red. In the third column, the designated code used to identify the different SA187-Flg peptides synthesized in this study. Four out of five peptides were used since peptide 4 and peptide 5 share the same amino acids composition in the selected 22-peptide lenght analyzed, for this reason they were indicated P4/P5 hereinafter. **(B)** Time course MAPK activation in Col-0 seedlings after treatment with 1 μM flagellin peptides encoded by SA187 in comparison to *P. syringae* Flg22 or mock inoculation for 15, 30 and 60 min. Comassie staining of the membrane was used to demonstrate similar protein loading in each well. M= protein marker. **(C)** Seedling growth inhibition assay. Five-day-old Col-0 and *fls2* seedlings were transferred to liquid MS supplemented or not with the different flagellin peptides at 100 nM concentration. Plants were weighted 7 days after treatment. Bars represent the average ± SD (*n = 12).* Statistical analysis was performed by applying the two-way ANOVA with Bonferroni’s test. ****: p≤ 0.0001, ***: p≤ 0.001, n.s: statistically non-significant.


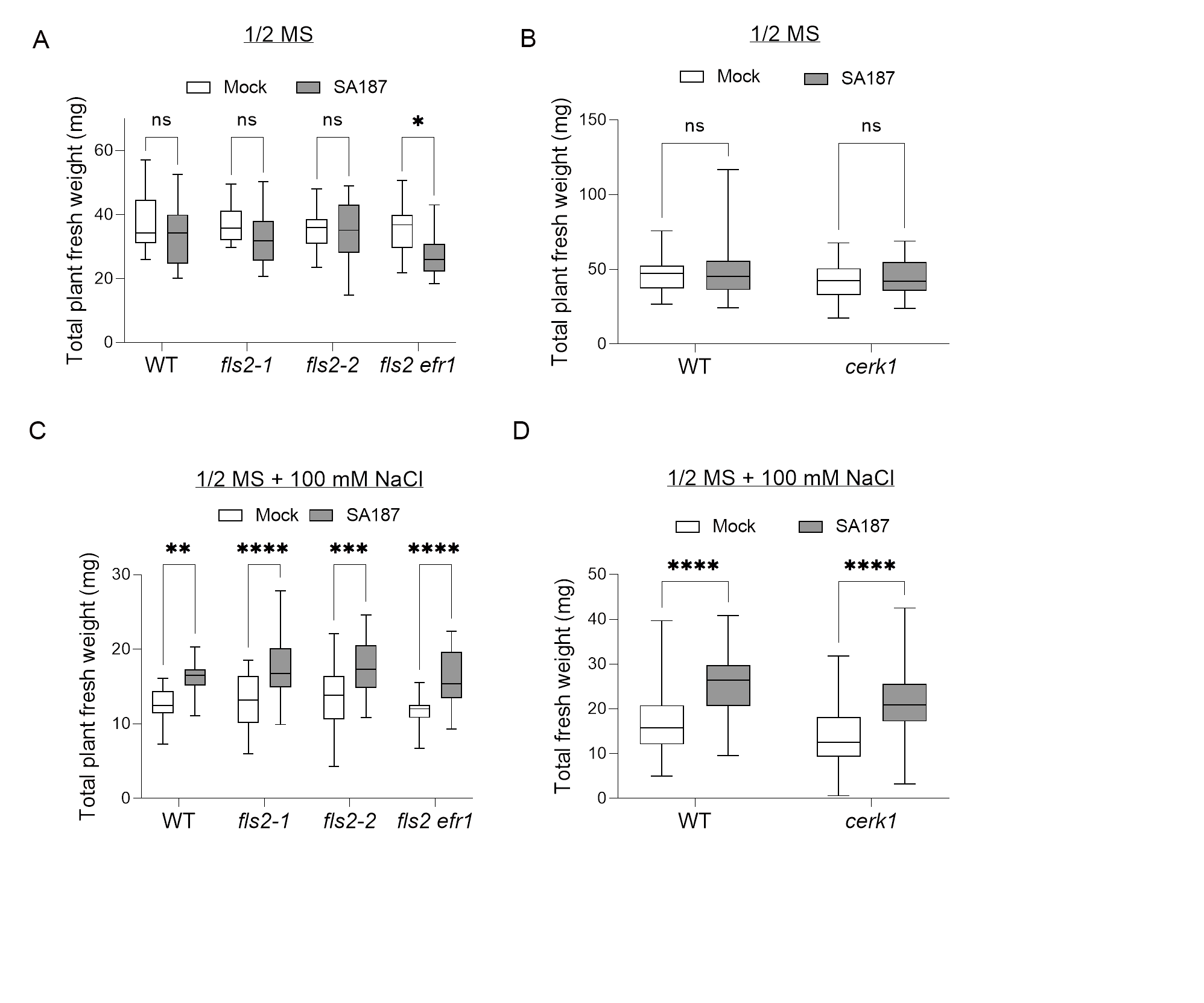


**SUPPORTING FIGURE 3. SA187–induced plant growth under control conditions (1/2 MS) and under salt stress (1/2 MS + 100mM NaCl) in Arabidopsis lines affected in *FLS2, EFR1,* and *CERK1.***

(A) SA187-induced plant growth under control conditions in two *fls2* mutant alleles and in *fls2 efr1* background. 24<N° of plants >28, data represented in the graph correspond to two independent experiments. (B) SA187-induced plant growth under control conditions in *cerk1* background. 58<N° of plants >72, data represented in the graph represent two independent experiments. (C) SA187-induced plant growth on Arabidopsis plantlets under salt stress in two *fls2* mutant alleles and in *fls2 efr1* background. 17<N° of plants >23, data represented in the graph represent two independent experiments. (D) SA187-induced plant growth of on Arabidopsis plantlets under salt stress in *cerk1* mutant. 55<N° of plants >68, data represented in the graph represent two independent experiments.

In the different graphs values are represented as average ± standard error. Statistical analysis was performed by applying the two-way ANOVA, comparing SA187 treated samples vs mock inoculation in each genetic background. * for p≤ 0,05; ** for p≤ 0,01; *** for p≤0,001; **** for p≤0,0001; ns: statistically non-significant.


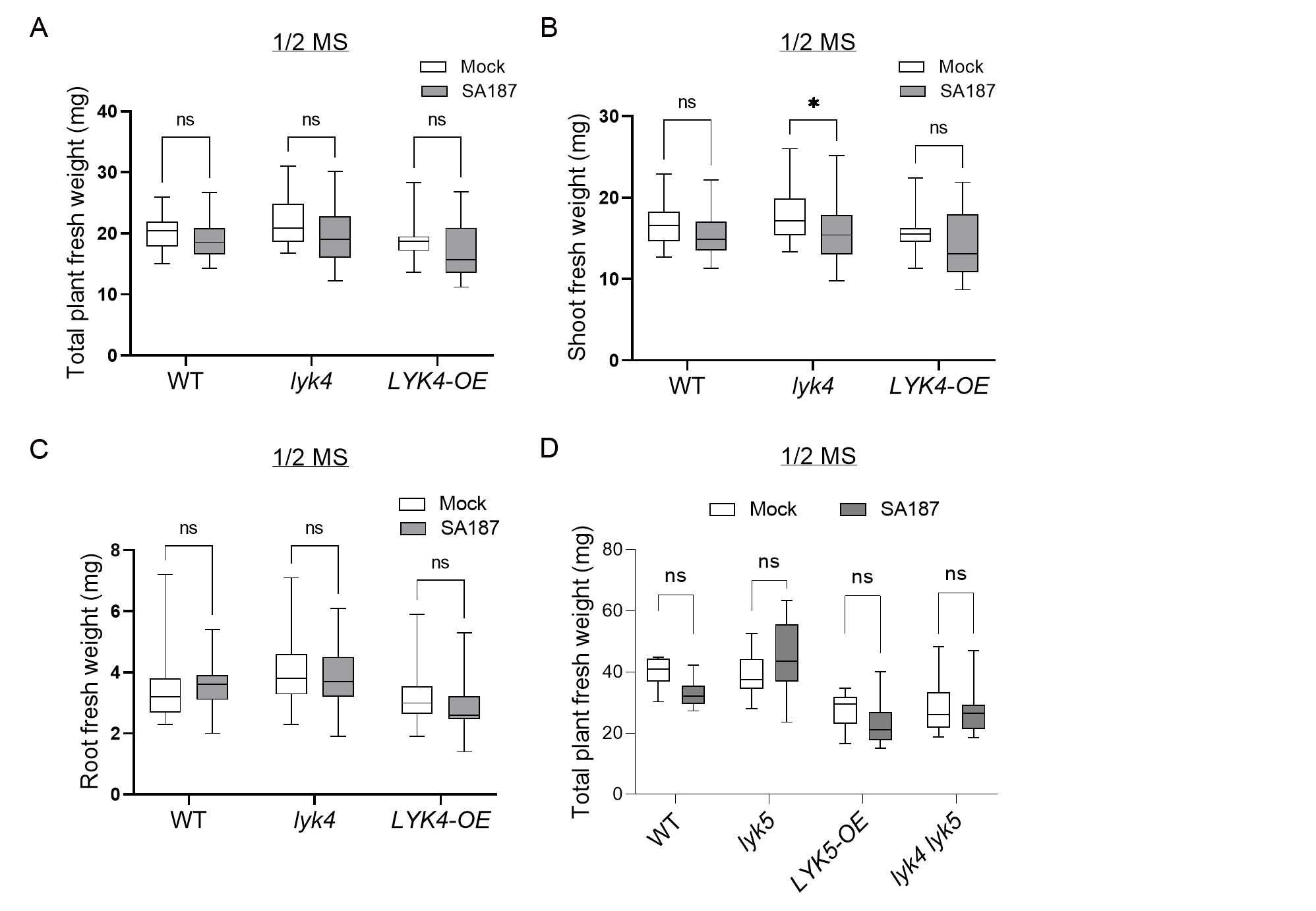


**SUPPORTING FIGURE 4. *Enterobacter* sp. SA187 poorly affected plant growth under control conditions (1/2 MS) in *lyk4*, *LYK4-OE*, *lyk5*, *LYK5-OE* and *lyk4 lyk5*.**

**(A)** Total plant fresh weight, **(B)** Shoot fresh weight and **(C)** Root fresh weight of mock and SA187-colonized plantlets under control conditions (1/2 MS) in WT, *lyk4* and *LYK4-OE* backgrounds. 66 ≤ N° plants analyzed ≥ 87 in three independent experiments. Statistical analysis was performed by applying the two-way ANOVA. * for p≤ 0,05; ns: statistically non-significant. **(D)** Total plant fresh weight of mock and SA187-inoculated plants under control conditions (1/2 MS) in *lyk5*, *LYK5-OE* and *lyk4 lyk5* backgrounds. N° of plants =33, data represented in the graphs represent three independent experiments. Statistical analysis was performed by applying the two-way ANOVA. ns: statistically non-significant


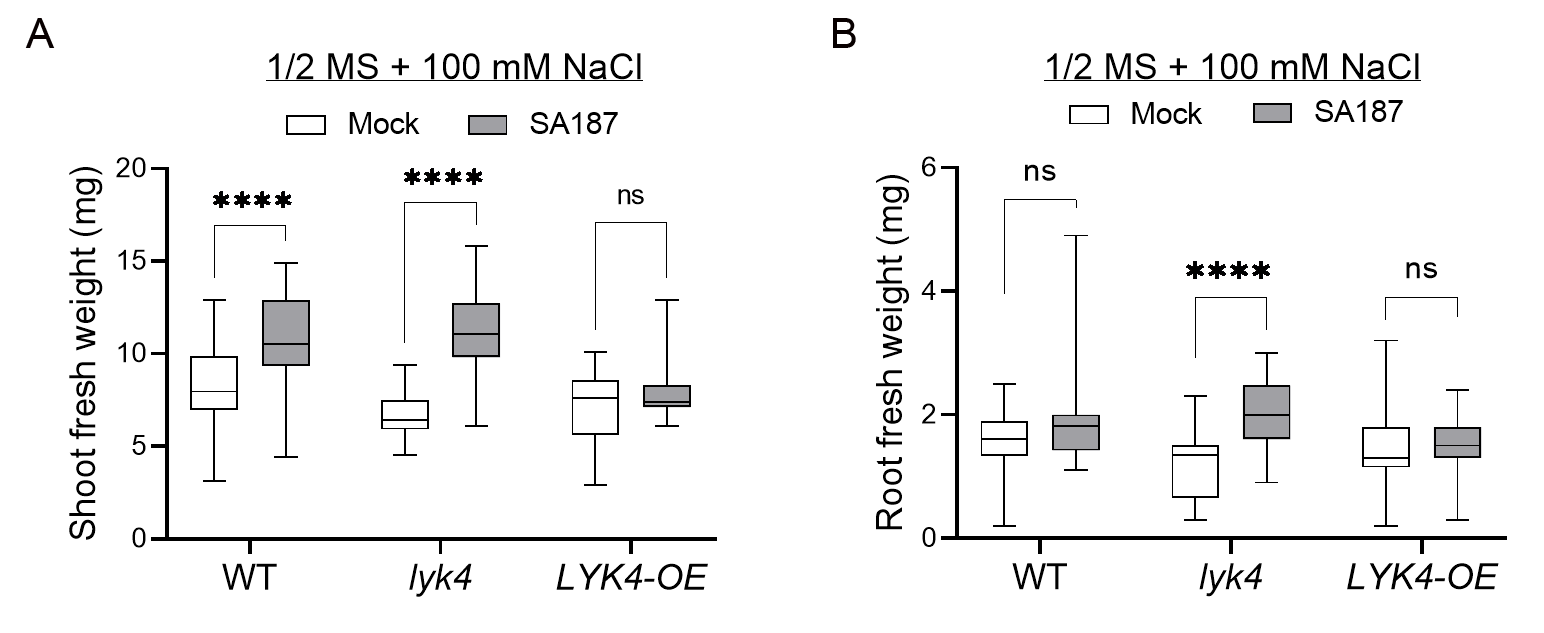


**SUPPORTING FIGURE 5. *Enterobacter* sp. SA187 affected plant growth under salt conditions (1/2 MS + 100mM NaCl) in *lyk4* and *LYK4-OE.***

**(A)** Shoot fresh weight and **(B)** Root fresh weight of mock and SA187-colonized plantlets under salt conditions (1/2 MS + 100mM NaCl) in WT, *lyk4* and *LYK4-OE* backgrounds. 25≤N° of plants≥36. Data represented in the graphs represent three independent experiments. Statistical analysis was performed by applying the two-way ANOVA, comparing SA187 treated samples vs mock inoculation in each genetic background. **** for p≤0,0001; ns: statistically non-significant.


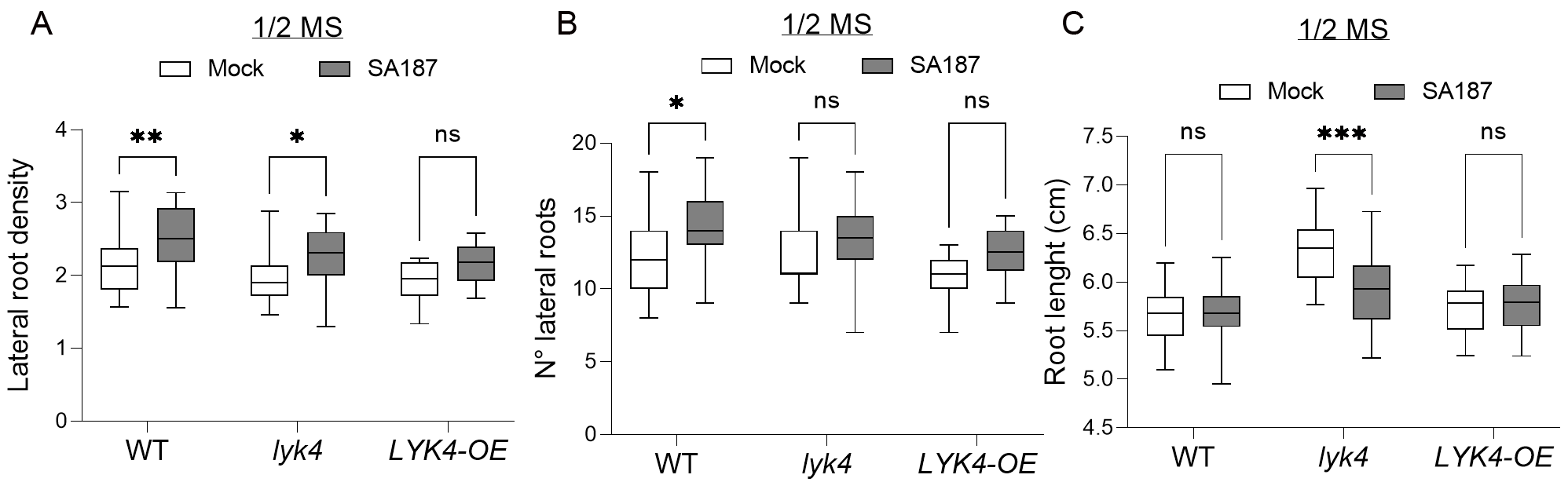


**SUPPORTING FIGURE 6. SA187-induced remodeling of root architecture under control conditions (1/2 MS) in WT, *lyk4* and *LYK4-OE*.** **(A)** Lateral root density, **(B)** N° of lateral roots and **(C)** Primary root length. N° plants analyzed = 24 in three independent experiments. Statistical analysis was performed by applying the two-way ANOVA, comparing SA187 treated samples vs mock inoculation in each genetic background. * for p≤ 0,05; ** for p≤ 0,01; *** for p≤0,001; ns: statistically non-significant.


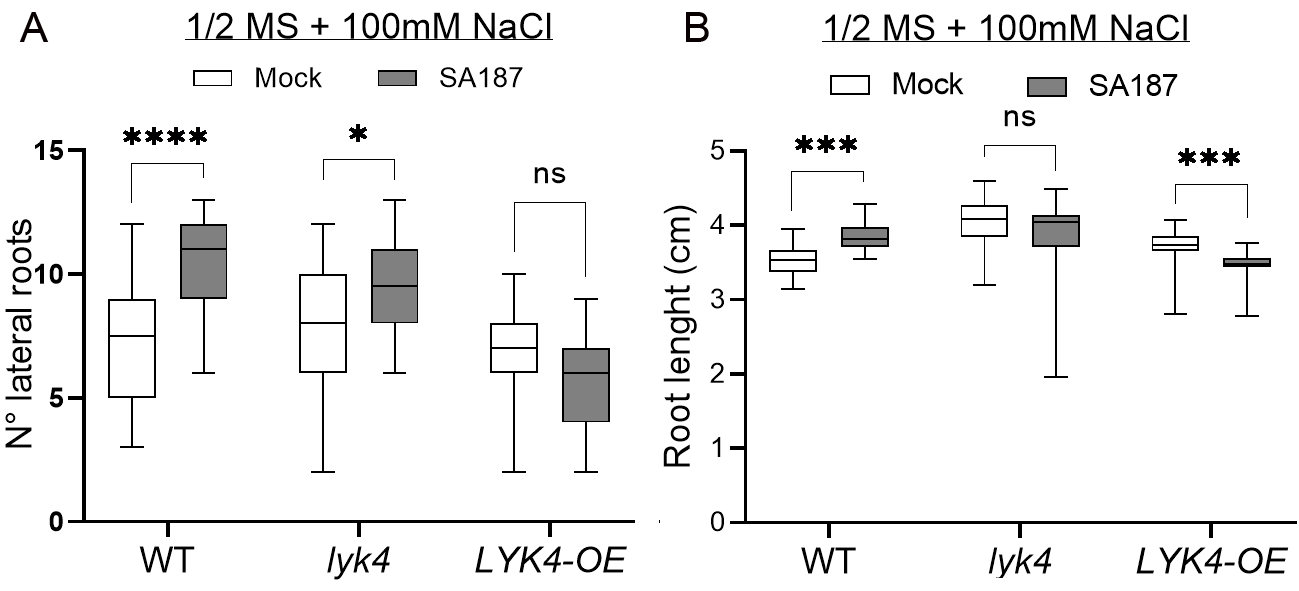


**SUPPORTING FIGURE 7. SA187-induced remodeling of root architecture under salt stress (1/2 MS + 100mM NaCl) in WT, *lyk4* and *LYK4-OE*.** **(A)** N° of lateral roots and **(B)** Primary root length. N° plants analyzed = 24 in three independent experiments. Statistical analysis was performed by applying the two-way ANOVA, comparing SA187 treated samples vs mock inoculation in each genetic background. * for p≤ 0,05; ** for p≤ 0,01; *** for p≤0,001; **** for p≤0,0001; ns: statistically non-significant.


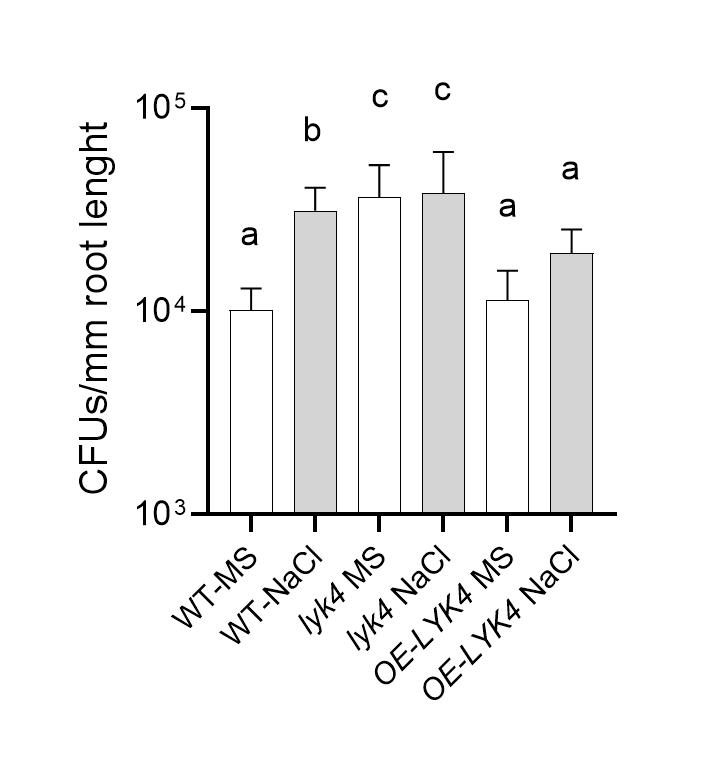


**SUPPORTING FIGURE 8. Colonization efficiency of SA187 on** **WT, *lyk4* and *OE-LYK4* plants.**

The colonization efficiency was evaluated through re-isolation of SA187 from WT, *lyk4* and *OE-LYK4* colonized plants grown for 3 days on ½ MS or ½ MS + 100 mM NaCl and was expressed as CFUs (colony forming units) per millimeter of primary root length. Statistical analysis was performed by applying one-way ANOVA with Tukey’s multiple comparison test. ** for p≤ 0,01; ns: statistically non-significant.


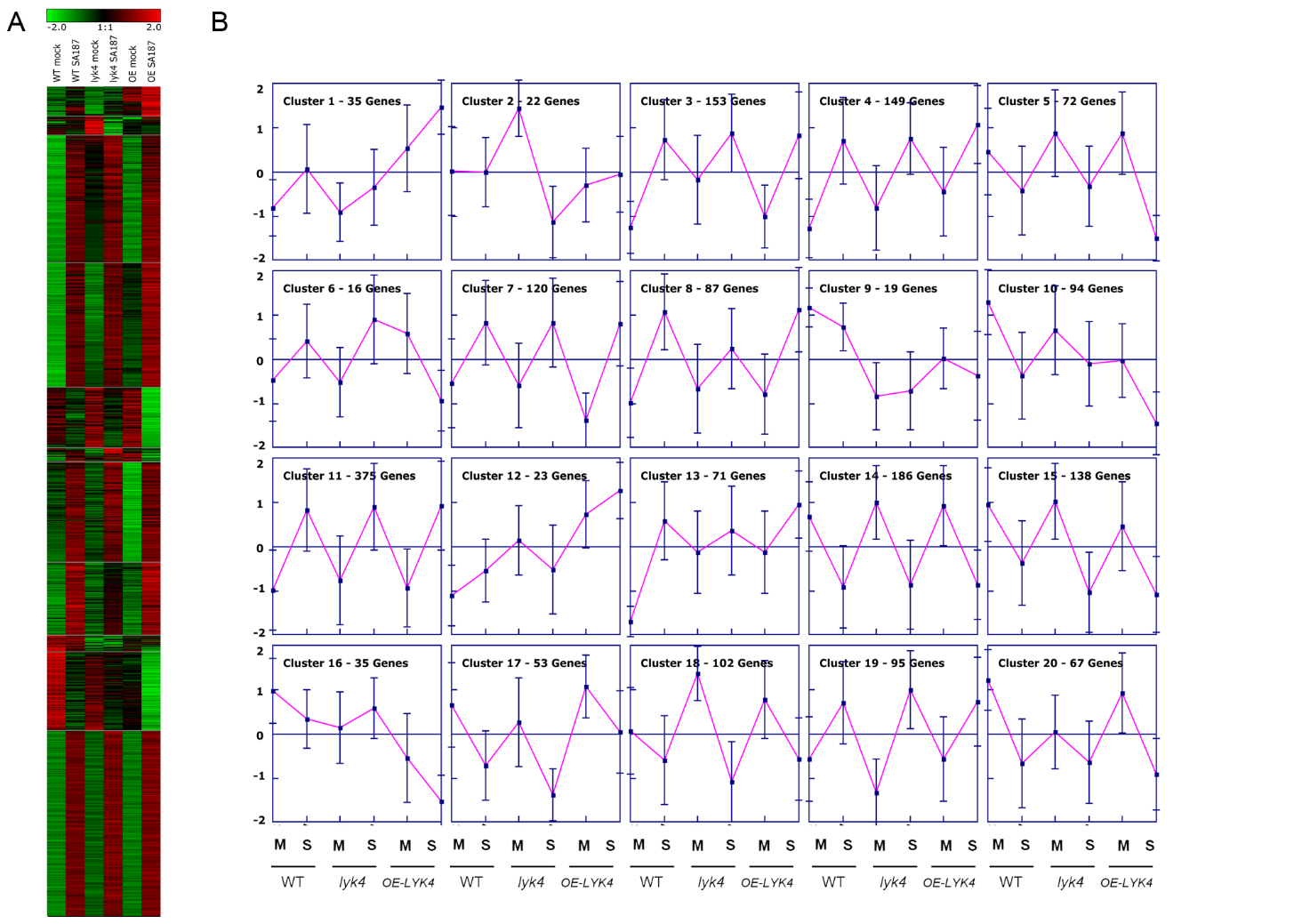


**SUPPORTING FIGURE 9. Transcriptomic analysis in WT, *lyk4* and *LYK4-OE* upon beneficial interaction with SA187 under non-salt conditions.**

(A) Hierarchical clustering of 1912 differentially expressed genes under non-salt conditions (1/2 MS) in WT, l*yk4* and *LYK4-OE* genotypes. (B) The 20 clusters in which the DEGs were organized according to their expression pattern.


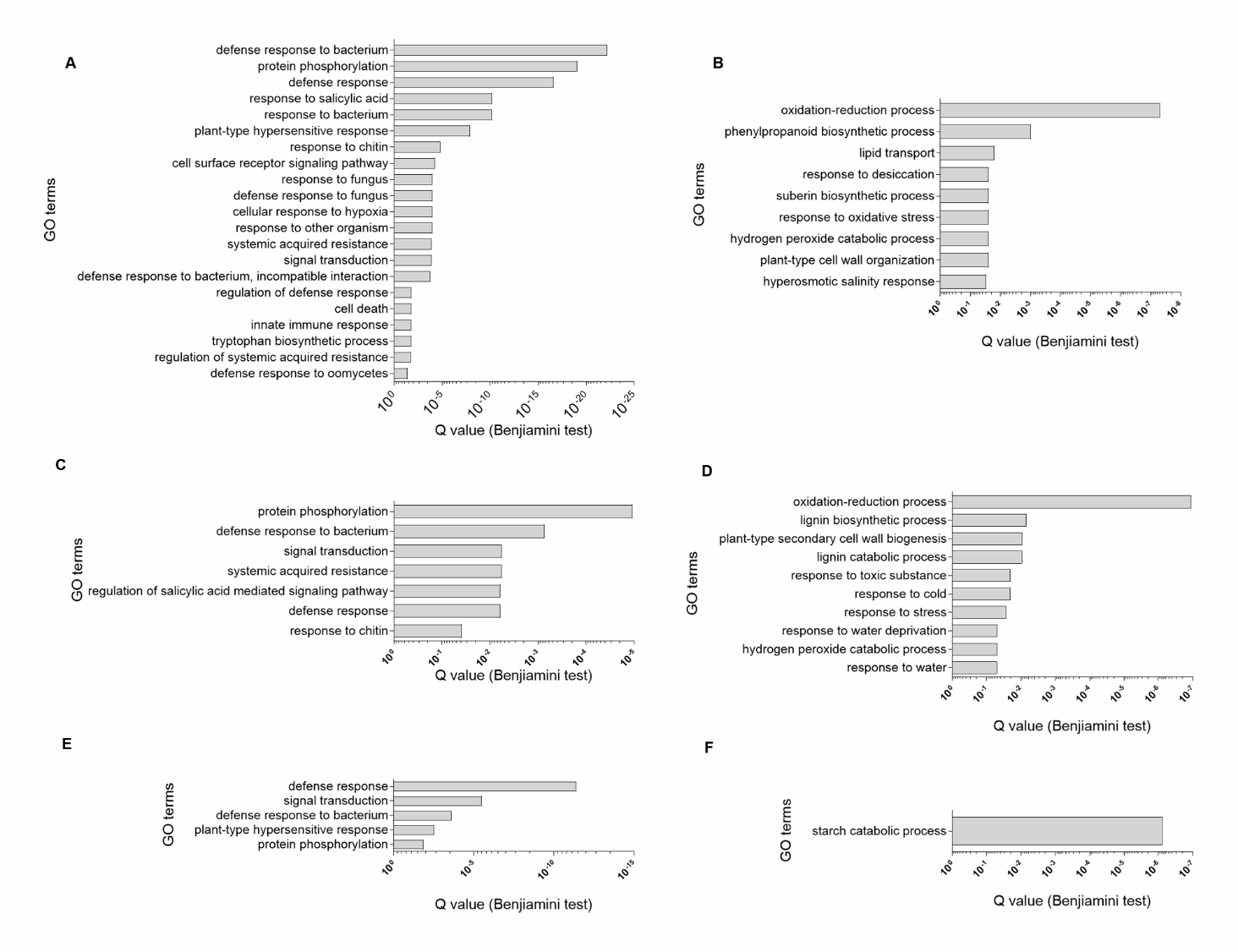


**SUPPLEMENTARY FIGURE 10. Gene Ontology terms of the main clusters for transcriptomic analysis comparing WT, *lyk4* and *LYK4-OE* under non-salt conditions on 1/2 MS medium according to DAVID database. (A)** GO terms in cluster 11; **(B)** GO terms in cluster 14; **(C)** GO terms in cluster 3; **(D)** GO terms in cluster 15; **(E)** GO terms in cluster 4; **(F)** GO terms in cluster 10.

**
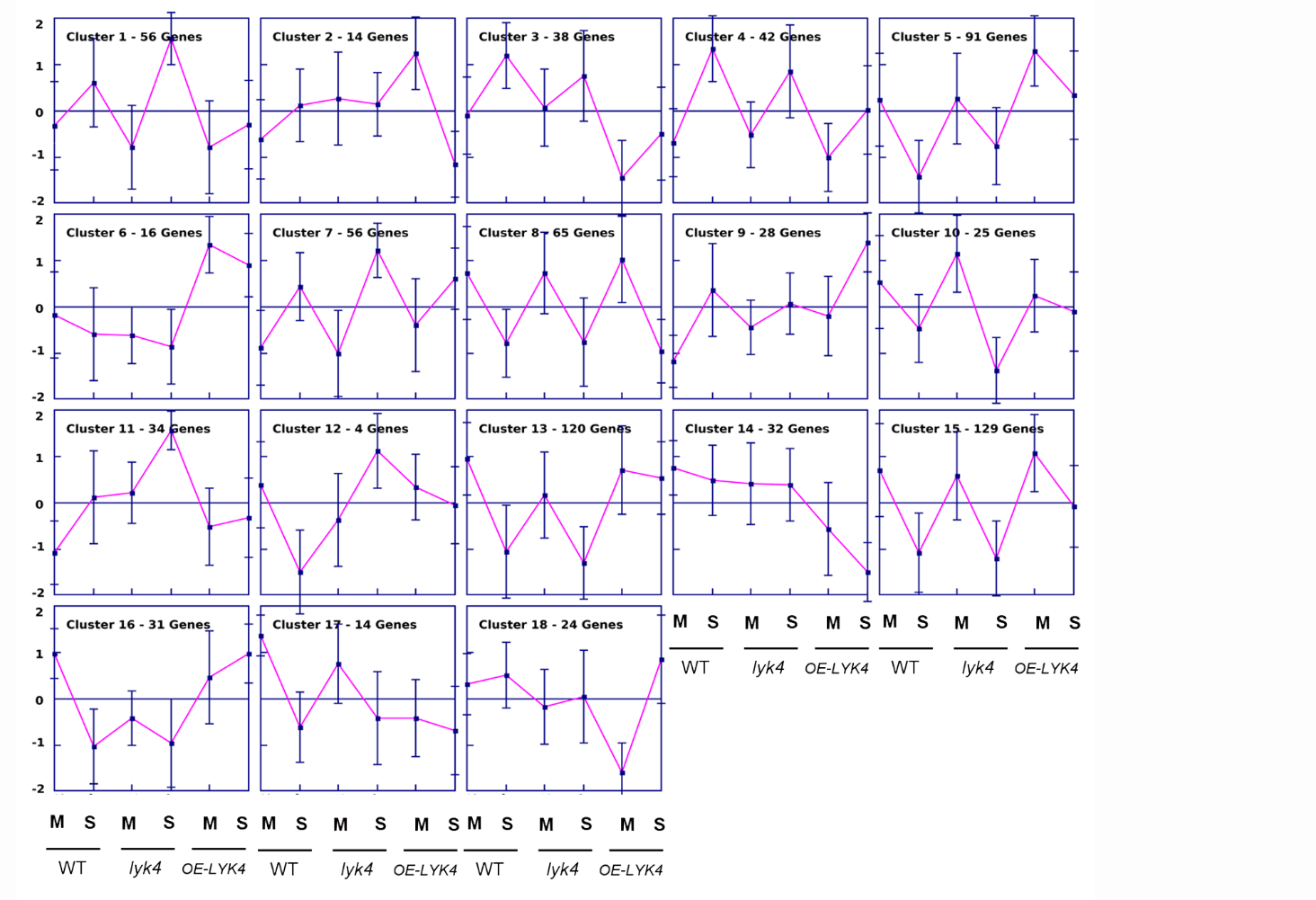
**

**SUPPORTING FIGURE 11. Expression pattern of the differentially expressed genes under salt stress conditions (1/2 MS + 100mM NaCl) in WT, *lyk4* and *LYK4-OE* genotypes**.

819 DEGs under salt stress conditions were organized by hierarchical clustering into 18 groups and the different expression patterns of genes in each cluster are reported in the different centroid graphs. M=mock inoculation; S= SA187-treated plantlets.


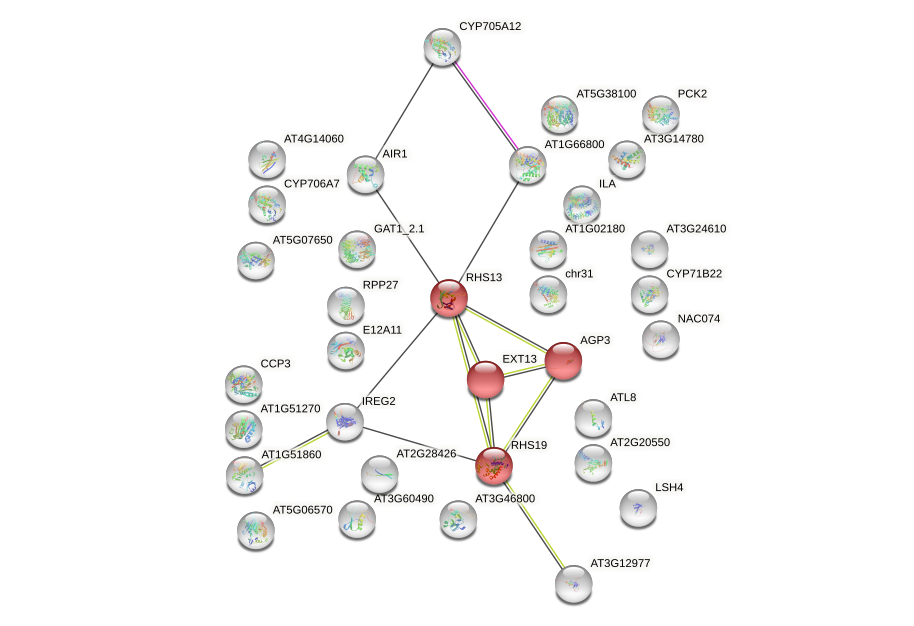


**SUPPORTING FIGURE 12. STRING analysis cluster 11 of transcriptome data analysis under salt stress conditions.** **(A)** Functional association network of proteins in cluster 11 according to STRING. The red color highlights that the indicated proteins belong to the GO term related to regulation of cell wall pectin metabolic process.


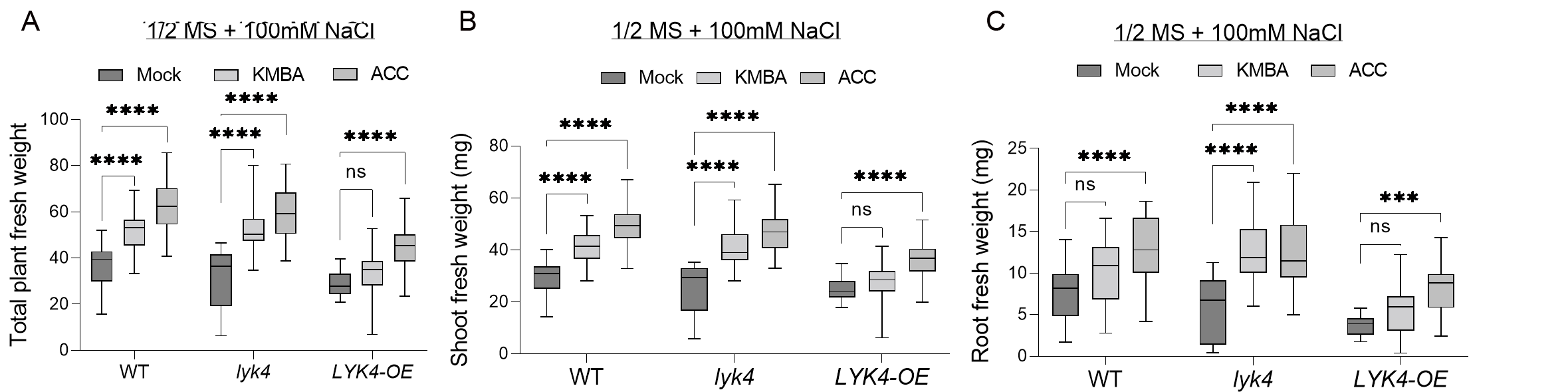


**SUPPORTING FIGURE 13. Role of *LYK4* in mediating salt stress tolerance induced by KMBA and ACC.**

(A) Total plant fresh weight, (B) Shoot fresh weight and (C) Root fresh weight of WT, *lyk4* and *LYK4-OE* plantlets under saline stress (1/2 MS + 100 mM NaCl) exposed to the ethylene precursors KMBA or ACC. Statistical analysis was performed by applying the two-way ANOVA, comparing SA187 treated samples vs mock inoculation in each genetic background. * for p≤ 0,05; ** for p≤ 0,01; *** for p≤0,001; **** for p≤0,0001; ns: statistically non-significant.


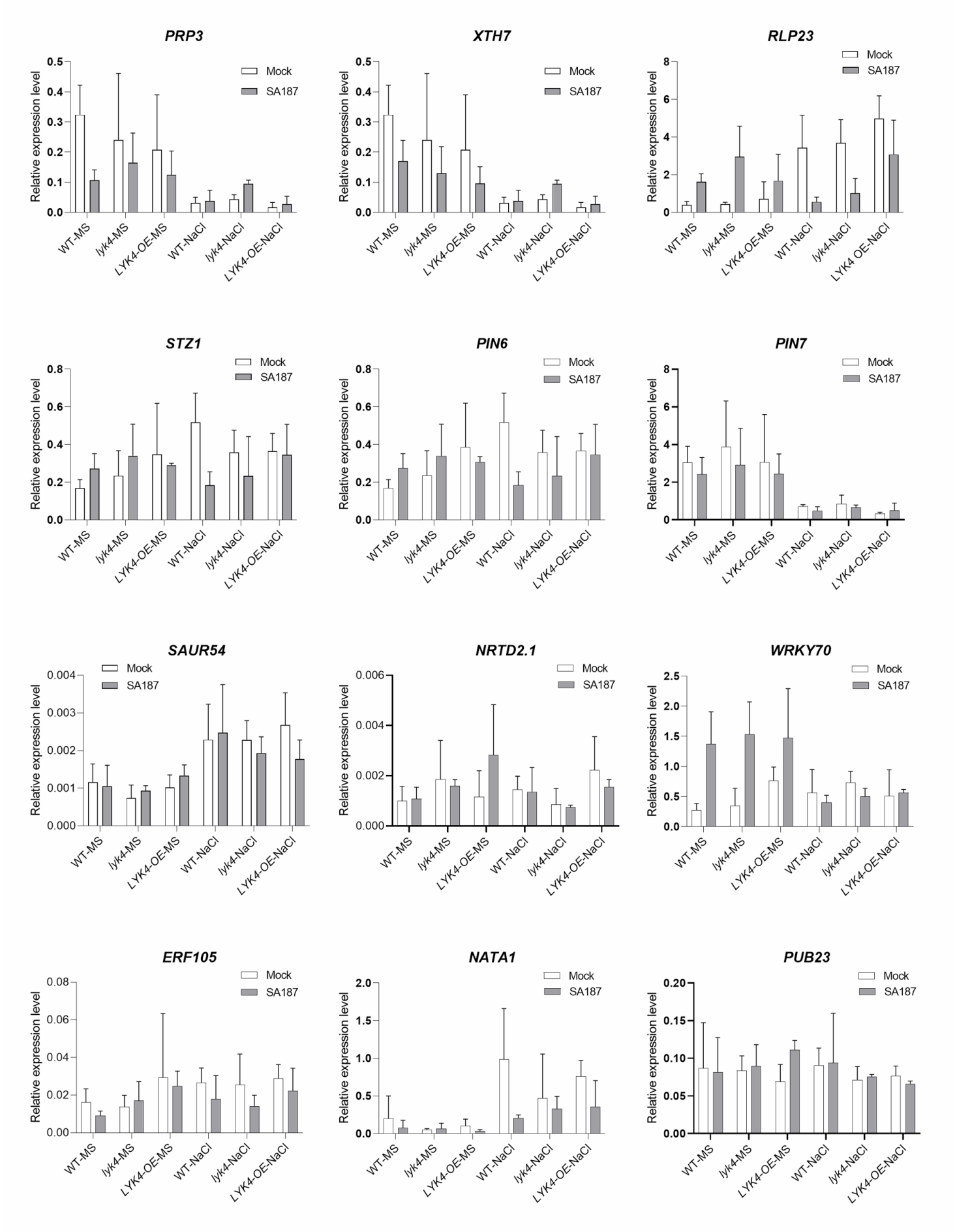


**SUPPORTING FIGURE 14**. qRT-PCR confirmation of RNA seq data.

**SUPPORTING TABLE 1.**

Beneficial index induced by SA187 in different PRR mutant backgrounds under salt stress. To calculate the beneficial index in each genetic background, the ratio between the plant fresh weigh of SA187- and mock- inoculated seedlings was calculated and expressed in percentage. The statistical analysis was performed by applying the Mann and Whitney test.

| **Genetic background** | **Beneficial index Replicate 1 (%)** | **Beneficial index Replicate 2 (%)** | **Beneficial index Replicate 3 (%)** | **Beneficial index average ±standard deviation (%)** | **Statistical analysis** |
| --- | --- | --- | --- | --- | --- |
| Wild type | 26.7 | 31.5 | n.a. | 29.1±3.3 | - |
| *fls2-1* | 41.2 | 30 | n.a. | 35.6±8 | n.s |
| *fls2-2* | 36.5 | 21.2 | n.a. | 28.9±10.8 | n.s |
| *fls2 efr1* | 53.5 | 30.9 | n.a. | 42.2±16 | n.s |
| Wild type | 41.1 | 39.5 | n.a. | 40.3±1.1 | - |
| *cerk1* | 69.4 | 34.1 | n.a. | 51.7±24 | n.s |
| Wild type | 31 | 32 | 38 | 38±3.7 | - |
| *lyk4* | 85 | 55 | 52 | 64±18 | p≤0.01 |
| *LYK4-OE* | 1.75 | 14.7 | 22 | 12±10.2 | p≤0.01 |
| Wild type | 40 | 30.2 | 46.9 | 39±8.3 | - |
| *lyk5* | 52 | 40 | 43.6 | 45±6.1 | n.s |
| *LYK5-OE* | 3.7 | 8.5 | 6.5 | 6.3±2.4 | p≤0.01 |
| *lyk4 lyk5* | 56 | 55 | 42.7 | 51.2±7.4 | n.s |

n.a: not available since two biological replicates were run.

n.s: non statistically relevant.

**SUPPORTING TABLE 2**

Beneficial index induced by KMBA in WT, *lyk4* and *LYK4-OE* under salt stress. To calculate the beneficial index in each genetic background, the ratio between the plant fresh weigh of SA187- and mock- inoculated seedlings was calculated and expressed in percentage. The statistical analysis was performed by applying the Mann and Whitney test.

| **Genetic background** | **Beneficial index Replicate 1 (%)** | **Beneficial index Replicate 2 (%)** | **Beneficial index Replicate 3 (%)** | **Beneficial index average ±standard deviation (%)** | **Statistical analysis** |
| --- | --- | --- | --- | --- | --- |
| Wild type | 45.08 | 48.08 | 23.02 | 38.7±13.6 | - |
| *lyk4* | 63.3 | 71.3 | 91.7 | 75.14±14.7 | p≤0.001 |
| *LYK4-OE* | 11.4 | 6.9 | n.a. | 9.17±3.1 | p≤0.01 |

n.a: not available since two biological replicates were run.

n.s: non statistically relevant.

**SUPPORTING TABLE 3. RNA-seq values for the investigated genes used in qRT-PCR validation.**

| **Gene** | **½ MS** | | | | | | **½ MS + 100mM NaCl** | | | | | |
| --- | --- | --- | --- | --- | --- | --- | --- | --- | --- | --- | --- | --- |
|  | **WT** | | ***lyk4*** | | ***LYK4-OE*** | | **WT** | | ***lyk4*** | | ***LYK4-OE*** | |
|  | **Mock** | **SA187** | **Mock** | **SA187** | **Mock** | **SA187** | **Mock** | **SA187** | **Mock** | **SA187** | **Mock** | **SA187** |
| ***PRP3*** | 17.8 | 13.6 | 18.5 | 13.1 | 15.9 | 11.6 | 1.5 | 2.8 | 2.5 | 4.1 | 2.2 | 2.2 |
| ***XTH7*** | 45.1 | 35.4 | 43.6 | 37.1 | 39.3 | 36.2 | 20.8 | 31.6 | 22.1 | 31.5 | 23.4 | 26.6 |
| ***RLP23*** | 1.8 | 18.9 | 3.7 | 31.8 | 1.6 | 21.5 | 17.3 | 4.4 | 25.2 | 8.2 | 40.5 | 24.5 |
| ***STZ1*** | 5.7 | 4.4 | 5.5 | 4.8 | 5 | 4.2 | 4.5 | 3 | 5.3 | 3.7 | 5.7 | 4.3 |
| ***PIN6*** | 0.9 | 1.4 | 1.4 | 0.7 | 1.2 | 1.3 | 0.6 | 0.8 | 0.7 | 1.6 | 0.6 | 1.5 |
| ***PIN7*** | 55.2 | 37.8 | 49.6 | 43.2 | 38 | 34.3 | 36.8 | 58.4 | 40.1 | 45.1 | 37.5 | 45.1 |
| ***SAUR54*** | 2.5 | 3.4 | 2.2 | 3.5 | 3.2 | 3.8 | 1.7 | 2.8 | 3.7 | 3 | 3.3 | 6.2 |
| ***NRTD2.1*** | 1.4 | 2.6 | 1.3 | 1.4 | 1.1 | 4.8 | 0.6 | 1.6 | 1.3 | 0.7 | 2.2 | 1.7 |
| ***WRKY70*** | 29.2 | 142.8 | 33.1 | 140.5 | 29.4 | 163.7 | 44.8 | 27.7 | 52.4 | 37 | 66.7 | 57.8 |
| ***ERF105*** | 9.2 | 7.8 | 7.9 | 8.6 | 6.7 | 10.6 | 7.1 | 6 | 9 | 7.4 | 2.2 | 1.7 |
| ***NATA1*** | 9.2 | 6.3 | 2.3 | 2.4 | 2.6 | 0.9 | 15.9 | 6.1 | 38.5 | 7.7 | 26.3 | 8.9 |
| ***PUB23*** | 11.7 | 21.9 | 15.3 | 21.2 | 11.6 | 22.5 | 8.5 | 13.6 | 9.7 | 11.3 | 11.6 | 12.7 |

**SUPPORTING MATERIAL 1. List of primers used in qRT PCR experiments.**

| **GENE** | **Primers** | **Reference** |
| --- | --- | --- |
| ***WRKY33*** | Farward: ACAACAGCAACCTTCAAACG  Reverse: ATCCTTTGGTGGCAGAAATG | **-** |
| ***FRK1*** | Farward: CGGTCAGATTTCAACAGTTGTC  Reverse: AATAGCAGGTTGGCCTGTAATC | (Boudsocq *et al.*, 2010) |
| ***PUB23*** | Farward:CAAGCGATAGAGCGGTTAGG  Reverse: CTCCTTTGCCTTCTCTTTGG |  |
| ***MYB51*** | Farward:TTAACCGCCACGGTGAAG  Reverse: TTGCCGCATCTCTTGAGTC |  |
| ***PRP3*** | Farward:GTTCCGACCCAGCATCATAC  Reverse: GCAAGTCTCGACCGGAGATA | (Bergonci *et al.*, 2014) |
| ***XTH7*** | Farward: TTGGTTCGACCCTTCTCGTG  Reverse: CCCTGATGGGCACATTGTCT | (de Zélicourt *et al.*, 2018) |
| ***RLP23*** | Farward: GCTCTGTGATGGTGCCTCTT  Reverse GAGAGGACCTTGATGCGGAG |  |
| ***STZ1*** | Farward: AGTCGAGCACTGGACAAAGG  Reverse: TAGCTCAACTTCTCCACCGC | (de Zélicourt *et al.*, 2018) |
| ***PIN6*** | Farward: TGGGCCGTTTTCTTCAAAGC  Reverse: GATTGATCCGGCTGCTTGAC | (Simon *et al.*, 2016) |
| ***PIN7*** | Farward: CCAAGATTAGTGGAACGCAAC  Reverse: GAAAAGGGTTTTTGGATCCTC | (Denoux *et al.*, 2008) |
| ***SAUR54*** | Farward: AAAGAAACAGAGCAGTGAGTATAAC  Reverse: TGGGAACATCCAGAGGAAGAG | (van Mourik *et al.*, 2017) |
| ***NRTD2.1*** | Farward: CCGACAAGACGGCCAAGTTCGACCT  Reverse: TCCGTAGAGAAGAACGAAGATCCAAG | (Okamoto *et al.*, 2003) |
| ***WRKY70*** | Farward: GAGGACGCATTTTCTTGGAG  Reverse: GCTCAACCTTCTGGACTTGC |  |
| ***ERF105*** | Farward: AAGCATGAGGACTTGGGAGA  Reverse: TTGATGGCGTTAATGGACAA |  |
| ***NATA1*** | Farward: GAGTCTGGTCTTGCCTCCAC  Reverse: ATGCGTCTCAAGAAAGGGGG | (Simon *et al.*, 2016) |
| ***ACTIN2*** | Farward: CGTTTCTATGATGCACTTGTGTG  Reverse: GGGAACAAAAGGAATAAAGAGG |  |

**Supporting Literature**

Bergonci, T., Ribeiro, B., Ceciliato, P.H.O., Guerrero-Abad, J.C., Silva-Filho, M.C., and Moura, D.S. (2014) Arabidopsis thaliana RALF1 opposes brassinosteroid effects on root cell elongation and lateral root formation. *J Exp Bot* **65**: 2219–2230.

Boudsocq, M., Willmann, M.R., Mccormack, M., Lee, H., Shan, L., He, P., et al. (2010) HHS Public Access. *Nature* **464**: 418–422.

Denoux, C., Galletti, R., Mammarella, N., Gopalan, S., Werck, D., De Lorenzo, G., et al. (2008) Activation of defense response pathways by OGs and Flg22 elicitors in Arabidopsis seedlings. *Mol Plant* **1**: 423–445.

van Mourik, H., van Dijk, A.D.J., Stortenbeker, N., Angenent, G.C., and Bemer, M. (2017) Divergent regulation of Arabidopsis SAUR genes: A focus on the SAUR10-clade. *BMC Plant Biol* **17**: 1–14.

Okamoto, M., Vidmar, J.J., and Glass, A.D.M. (2003) Regulation of NRT1 and NRT2 gene families of Arabidopsis thaliana: Responses to nitrate provision. *Plant Cell Physiol* **44**: 304–317.

Simon, S., Skůpa, P., Viaene, T., Zwiewka, M., Tejos, R., Klíma, P., et al. (2016) PIN6 auxin transporter at endoplasmic reticulum and plasma membrane mediates auxin homeostasis and organogenesis in Arabidopsis. *New Phytol* **211**: 65–74.

de Zélicourt, A., Synek, L., Saad, M.M., Alzubaidy, H., Jalal, R., Xie, Y., et al. (2018) Ethylene induced plant stress tolerance by *Enterobacter* sp. SA187 is mediated by 2‐keto‐4‐methylthiobutyric acid production. *PLoS Genet* **14**: 1–28.

**SUPPORTING FILES**

**SUPPORTING FILE 1.**

The excel file contains the information about hierarchical clusters and the main GO associated terms for the 1912 DEGs that are differentially expressed among mock and SA187 treated WT, *lyk4* and *OE-LYK4* under control conditions (1/2 MS).

The sheet named “all clusters” contains the list of DEGs in each cluster.

The sheets named “3”, “4”, “7”, “10”, “11”, “14”, “15”, “18” contain the information about the GO associated term to each cluster according to DAVID database.

**SUPPORTING FILE 2.**

The excel file contains the information about hierarchical clusters and the main GO associated terms for the 819 DEGs that are differentially expressed among mock and SA187 treated WT, *lyk4* and *OE-LYK4* under salt stress (1/2 MS + 100 mM NaCl).

The sheet named “all clusters” contains the list of DEGs in each cluster.

The sheets named “1”, “3”, “5”, “7”, “8”, “13”, “15” contain the information about the GO associated term to each cluster according to DAVID database.
